# Enhancing microbial predator–prey detection with network and trait-based analyses

**DOI:** 10.1186/s40168-025-02035-8

**Published:** 2025-02-04

**Authors:** Cristina Martínez Rendón, Christina Braun, Maria Kappelsberger, Jens Boy, Angélica Casanova-Katny, Karin Glaser, Kenneth Dumack

**Affiliations:** 1 Terrestrial Ecology, Institute of Zoology, University of Cologne, Zülpicher Str. 47B, 50674 Cologne, Germany; 2 Institute for Biosciences, TU Bergakademie Freiberg, Leipziger Str. 29, Freiberg, Germany; 3 Institute of Ecology and Evolution, Friedrich Schiller University Jena, Dornburger Str. 159, 07743 Jena, Germany; 4 Institute of Planetary Geodesy, Technical University of Dresden, Helmholtz Str. 10, 01069 Dresden, Germany; 5 Institute of Earth System Sciences, Leibniz Universität Hannover, Herrenhäuser Str. 2, 30419 Hannover, Germany; 6 Department of Environmental Sciences, Faculty of Natural Resources, Catholic University of Temuco, Manuel Montt 56, Temuco, Chile

**Keywords:** Cross-kingdom network analyses, Trait-based ecology, Predator–prey interactions, Biocrusts, Microbial ecology, Microbial communities, Experimental validation

## Abstract

**Background:**

Network analyses are often applied to microbial communities using sequencing survey datasets. However, associations in such networks do not necessarily indicate actual biotic interactions, and even if they do, the nature of the interactions commonly remains unclear. While network analyses are valuable for generating hypotheses, the inferred hypotheses are rarely experimentally confirmed.

**Results:**

We employed cross-kingdom network analyses, applied trait-based functions to the microorganisms, and subsequently experimentally investigated the found putative predator–prey interactions to evaluate whether, and to what extent, correlations indicate actual predator–prey relationships. For this, we investigated algae and their protistan predators in biocrusts of three distinct polar regions, i.e., Svalbard, the Antarctic Peninsula, and Continental Antarctica. Network analyses using FlashWeave indicated that 89, 138, and 51 correlations occurred between predatory protists and algae, respectively. However, trait assignment revealed that only 4.7–9.3% of said correlations link predators to actually suitable prey. We further confirmed these results with HMSC modeling, which resulted in similar numbers of 7.5% and 4.8% linking predators to suitable prey for full co-occurrence and abundance models, respectively. The combination of network analyses and trait assignment increased confidence in the prediction of predator–prey interactions, as we show that 82% of all experimentally investigated correlations could be verified. Furthermore, we found that more vicious predators, i.e., predators with the highest growth rate in co-culture with their prey, exhibit higher stress and betweenness centrality — giving rise to the future possibility of determining important predators from their network statistics.

**Conclusions:**

Our results support the idea of using network analyses for inferring predator–prey interactions, but at the same time call for cautionary consideration of the results, by combining them with trait-based approaches to increase confidence in the prediction of biological interactions.

Video Abstract

**Supplementary Information:**

The online version contains supplementary material available at 10.1186/s40168-025-02035-8.

## Background

Microbial life thrives through complex ecological interactions [1]. Depending on the context and the environmental factors, microorganisms engage in a broad variety of trophic or non-trophic interactions, positive, negative, or neutral, which can dynamically shift between cooperation, competition, antagonism, and exploitation [2–5]. Disentangling the underlying dynamics shaping community structure, assembly, and function, as well as the impact of the environment on the microbial communities, requires an understanding of microbial interactions [6]. Towards this goal, association network analysis provides a robust analytical tool to investigate and predict microbial interactions [7]. Network analyses are often applied to diverse microbial communities using datasets derived from high-throughput sequencing. These analyses statistically correlate abundance-based associations, which are visualized into a network, with nodes representing microorganisms and edges representing positive or negative associations between taxa [8]. However, networks only generate hypotheses about community structure and are not equivalent to ecological networks, i.e., the calculated associations might not accurately reflect true ecological interaction [8].

Network interpretation is a complex process that requires the careful consideration of several factors, such as data type (cross-sectional or time series), environmental parameters, and potential confounding factors (indirect interactions or similar niche requirements) [9, 10]. Due to the lack of comprehensive datasets detailing known microbial interactions with which network analyses can be finetuned, experimental validation through co-culturing is pivotal to ensure the accuracy of the network models [1, 11]. However, most network constructions are rarely followed by experimental confirmation [9].

Network validation has been explored in only a few studies using various experimental co-culture approaches. Jiang et al. [12] found that positively associated taxa pairs in co‑occurrence networks implied relationships such as neutralism, competition, and mutualism, varying with bacterial combination and cultivation time. Moreover, Jiang et al. [13] observed that neutralism predominated in 65.6% and 35.7% of positive and negative tested network associations, respectively, and that positive relationships in gut microbial communities, which might be attributed to the exchange of amino acids, short-chain fatty acids, and vitamins, are overestimated. Durán et al. [14] examined interkingdom microbial competition within the *Arabidopsis thaliana* root microbiome, finding antagonistic interactions by assembling synthetic communities. Yet network validation studies remain scarce mainly due to the intrinsic complexity of both the ecological systems tested and the calculated networks, as well as the labor-intensive nature of microbial isolation, particularly for fastidious and difficult-to-culture microorganisms [1, 12].

The selection of a suitable ecological system for network validation is fundamental, as it determines the feasibility of experimental manipulation and whether the system may be extended to broader ecological theories. Terrestrial environments harbor highly diverse microbiomes, including members of all three domains of life and viruses, all of which play key roles in regulating soil ecosystem processes [15]. Given the heterogeneous nature of the soil matrix, where interactions among microorganisms and with their environment remain largely obscured [7, 10], these ecosystems present both unique opportunities and challenges for exploring microbial network functions. Among terrestrial ecosystems, biological soil crusts (biocrusts) have emerged as suitable and valuable model systems for advancing ecological theories and understanding ecosystem functioning [16]. Biocrusts are superficial micro-ecosystems composed of soil particles and various proportions of eukaryotic and prokaryotic photoautotrophs and heterotrophs [17–19]. Polar biocrusts, in particular, exhibit a considerably lower complexity than many other microbial communities due to the extreme environmental conditions that drastically reduce biodiversity. They predominantly feature microbial primary producers and consumers with short life cycles, thus offering exceptional potential to study the drivers and functioning of microbial communities [20–22]. Additionally, biocrust taxa are relatively easy to culture and transport, which enables the construction of custom communities in small experimental units [23]. Hence, polar biocrusts provide a relatively simple food-web structure, making them an appropriate system for validating network hypotheses.

Nonetheless, even the analysis of low-complex systems could generate hundreds to thousands of putative correlations, raising the question: Where to start testing? Simplified networks can be attained by reducing network density through stricter corrected *p*-value thresholds for inferred edges or by increasing cutoffs for association strength, prevalence, or abundance filtering [24, 25]. However, applying overly strict criteria can result in omitting potentially meaningful interactions, which could lead to a less comprehensive network [24]. Prior research has employed serial dilutions to reduce the complexity of microbial communities, generated in silico networks on the reduced communities, and experimentally validated microbial pairs [12, 13]. However, these simplified communities may overlook important high-order interactions that, in natural conditions, are modulated by other species [12]. A promising alternative approach is to agglomerate taxa based on taxonomic or functional groups [8]. For instance, Lima-Mendez et al. [26] adopted a trait-based approach by grouping taxa into functional sets and validated network-generated hypotheses through microscopy, confirming symbiotic relationships based on a literature-curated collection of 574 known symbiotic interactions in marine eukaryotic plankton. Building on this concept, applying an ecological trait-based approach, which incorporates organism-specific traits (measurable features of an individual that potentially affect its fitness [27]) for network evaluation, could enhance the identification and validation of meaningful interactions. Trait-based ecology aims to link species diversity and traits with the identification of the underlying mechanisms controlling community structure and ecosystem functioning [28, 29].

**Fig. 1:**
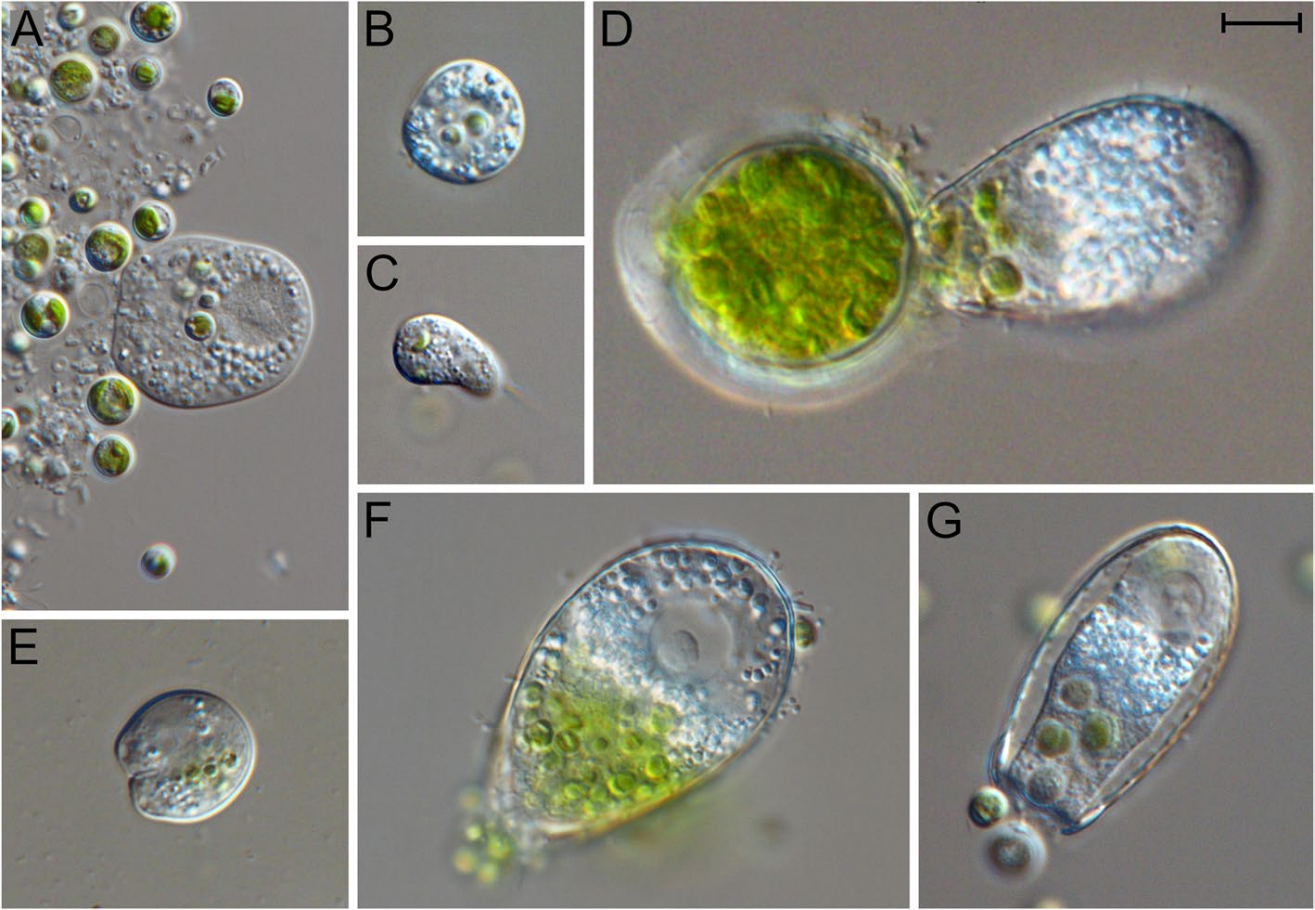
Selected images of algivorous Cercozoa and their prey covered in this study.** A** *Rhogostoma* sp. with ingested cells of *Auxenochlorella* sp. **B** *Fisculla terrestris* with two ingested cells of *Auxenochlorella* sp. **C** *Cercomonas celer* with food vacuoles containing Chlorellales cells. *Euglypha rotunda* (**D**, **F**, **G**) with food vacuoles containing **D** *Leptosira* sp.; **F **an undetermined Chlorellales; **G** *Bracteacoccus* sp. **E** *Rhogostoma* sp. with food vacuoles containing *Leptosira* sp. cells. The scale represents 10 µm

Here, we applied cross-kingdom network inference in polar biocrusts using DNA-based sequencing data to predict putative predator–prey relationships between cercozoans (Rhizaria) and their potential algal prey: green algae (Archaeplastida) and ochrophytes (Heterokontophyta). These taxa can dominate in terrestrial environments in both polar regions and play key roles in nutrient cycling and food-web dynamics [17, 30, 31]. Cercozoa are probably among the most common predators of terrestrial algae in polar biocrusts, as they can dominate among heterotrophic protists in biocrusts [32] and make up the majority of known terrestrial algivorous protists in other terrestrial environments (Fig. 1) [33, 34]. We developed taxon-specific DNA-based amplicon sequencing methods for green algae and ochrophytes to produce comprehensive datasets for Cercozoa and their putative prey. With the network analyses, we predicted predator–prey interactions, which we experimentally confirmed via food range experiments of here established cultures. We show the advantage of supplementing network analyses with the assignment of functional traits to increase confidence in predator–prey prediction and further show a promising correlation between node statistics and predator growth rates.

## Materials and methods

### Study areas and sample collection

This study encompasses the analysis of biological soil crusts in three polar regions. In the Arctic, one region was included — Svalbard in the Arctic Ocean (78° N). In Antarctica, two regions were studied, i.e., King George Island (62° S) in the South Shetland archipelago of maritime Antarctica and the Thala Hills oasis (67° S) in Enderby Land, East Antarctica. A brief description of the sampling sites is given below, and detailed information about their geographic location and climate is provided in Supplementary 1.

Sites with early and mature stages of biocrust development were selected. At each site, five replicates were collected, ensuring a minimum separation of 1 m. Sampling consisted of pressing the opening of a sterile plastic Petri dish into each biocrust and lifting it gently with a spatula disinfected with 70% ethanol. Next, the samples were air-dried for 1 to 3 days before sealing the Petri dishes with parafilm. Finally, the biocrusts were transported to Cologne, Germany, for analysis.

### Svalbard

Sampling sites comprised glacial moraines and other rocky terrains with sparse vegetation, where bird feces were present. Various bird species, including terns, skuas, and ptarmigans, were observed nesting in the area. The sampling campaign took place in July 2021. Forty-five soil crust samples were collected at 9 sampling sites in the vicinity of Longyearbyen. Three sites west of Longyearbyen, four in the periglacial area of Longyearbyen glacier, and three on the Breinosa mountain were sampled.

### King George Island

Sampling took place from January to March 2022. Fifty soil crust samples were collected in 10 sampling sites, chosen in areas with low human disruption. The sites featured Arenosols, Cryosols, Leptosols, and Fluvisols [35], which had bird droppings from the various species of skuas, gulls, terns, petrels, and penguins that visit or nest in the area. Three sites were located in the vicinity of Collins refuge and three in the *Meseta*, North Davis Heights, all of them in the periglacial area of Collins. Four additional samples originated from areas ranging from 300 m to 2 km in distance from Bellingshausen Station, on the Fildes Peninsula and Ardley Island in the south-west of King George Island.

### Thala Hills oasis, Enderby Land

The sampled terrain comprised rocky hills with weathered rock formations, occasionally accumulating bird droppings (Adélie penguins and south polar skuas nest in the area). The sampling campaign was conducted in February 2022 during the expedition PS128 [36] of the RV Polarstern [37]. Twenty soil crust samples were collected at four sampling sites located between 700 m to 3.2 km in the vicinity of the Molodezhnaya Station.

### Chemical properties of soil crusts

Total organic carbon (TOC), total nitrogen (TN), total phosphorus (TP), and pH were determined as described by Khanipour Roshan et al. [38]. Shortly, TN and TOC were measured with CNS Analyzer after treatment with HCl (Vario EL III, Elementar Analysensysteme, Hanau, Germany). TP was measured photometrically using molybdan blue as a color indicator. The pH was measured in a CaCl_2_ solution (1:2.5 ratio). Results are available in the Table S16.

### DNA extraction of soil crusts

Surface Sects. (2–3 mm) of each biocrust were separated from the adhering soil using a razor blade, until reaching a weight of 0.15 to 0.30 g. DNA was extracted using the Quick*-*DNA™ Fecal/Soil Microbe Midiprep kit (Zymo Research, Irvine, CA, USA) according to the manufacturer’s instructions. A FastPrep®−24 bead beater (MP Biomedicals, USA), equipped with a 2-ml tube holder assembly, was used for the bead-beating process, which entailed a single cycle at 4.5 m/s for 20 s. The kit includes a cleaning and concentration step, and the final elution volume was 100 μl.

### PCR amplification, library preparation, and sequencing

We aimed to employ taxon-specific metabarcoding approaches to obtain saturated data while reducing sequencing costs substantially, as with such a protocol all samples could be pooled and processed in one sequencing run per taxon. While there is a protocol available to study the Cercozoa in such a manner [39], we developed new protocols for both algal groups, which can be accessed in Supplementary 2. Briefly, after the design and successful testing, amplicons were generated using a semi-nested PCR approach. PCR mixtures of 11 and 17 μl were employed for the first and second PCR, respectively. The final concentrations for all three metabarcoding protocols were as follows: 0.01 units of DreamTaq Green DNA Polymerase and 1 × DreamTaq Green Buffer (Thermo Fisher Scientific, Dreieich, Germany), 0.2-mM dNTPs, and 1 μM of each primer. One nanogram of extracted DNA was incorporated on the first PCR, and 1 μl of the resulting amplicons was used as a template for the second. The amplification conditions for the three protocols initiated with denaturation at 95 °C (2 min), followed by 24 cycles of the three-step process of denaturation (95 °C, 30 s), annealing (Supplementary 2, Table S7; 30 s), and elongation (72 °C, 30 s), and concluded with an elongation step at 72 °C (5 min). After the second PCR, successful amplification and correct PCR product size were checked with an electrophoresis gel.

Amplicons, including internal standards (Supplementary 3), were purified and normalized using the SequalPrep Normalization Plate Kit (Invitrogen GmbH, Karlsruhe, Germany), to achieve a concentration of 1–2 ng/µl per sample, and finally pooled. Sequencing was performed by the Cologne Center for Genomics (Cologne, Germany) on an Illumina MiSeq platform (Illumina Inc., San Diego, CA, USA). Using the v3 reagent kit, and 2 × 300 cycles, 300-bp-long paired-end reads were produced.

### Sequence processing

Reads processing, as described by Fiore-Donno et al. [40], can be summarized as follows: raw reads underwent a quality check with the FastX toolkit (v. 0.0.13) [41]. Contigs were assembled by pairing reads using mothur (v.1.45.3) [42], allowing no differences in the primer sequences. Sequences with minimal overlap of 200 bp and a minimum length of 290 bp for green algae and ochrophytes, and 300 bp for Cercozoa, were selected, and those with ambiguities and more than one mismatch were removed. Sequences were demultiplexed via the detection of their unique primer adapters, which were then removed from the reads. Subsequently, and before conducting read clustering for the samples, the internal standard was analyzed and used to define filtering thresholds for clustering and denoising. According to those results, sequences were clustered into operational taxonomic units (OTUs) in mothur, using the abundance-based greedy clustering (AGC) of VSEARCH [43], and a similarity threshold of 97%. Next, the sequences were taxonomically assigned with the PR2 database [44] using BLAST + (v. 2.2.31) [45] with an *e*-value of 1e-50, and only the best hit was kept. Nontargeted taxa were removed, except for Endomyxa and Chrysophyceae, which were included in the downstream analyses of their respective datasets. The latter included the removal of two OTUs representing streptophytic microalgae (*Interfilum* sp. and *Cylindrocystis* sp.), which were subsequently excluded from further analysis. Cercozoan reads were then aligned to reference alignments [39]. Green algal and ochrophyte reference alignments comprise 150 representative sequences originating from the Diat.barcode database (v. 9) [46] and 399 sequences from the PR2 database, respectively. Both alignments were made with MAFFT (v. 7.221) using the L-INS-I algorithm (gap opening penalty = 3) [47]. Next, using the reference alignments, replicated sequences and chimeras were identified, with the latter being detected through the implementation of UCHIME [48] in mothur. Misaligned, replicated, and chimeric sequences were subsequently filtered out from the dataset. Analyses of the internal standard revealed that OTUs represented by fewer than 250 (Cercozoa), 305 (green algae), and 285 (ochrophytes) reads, and occurring in less than 3samples, were considered background noise and were removed accordingly. The final OTU counts served as the basis for downstream analyses, encompassing the calculation of diversity indexes, network analyses, and the confirmation of predation as a key functional trait of heterotrophic protists in polar biocrusts.

### Network inference

Putatively biotic interactions were detected and visualized via the calculation of cross-kingdom co-occurrence and correlation networks among the three investigated taxa. The analysis aimed to infer the role of cercozoan predation in shaping microalgae communities. To achieve this, we employed two complementary approaches:

### FlashWeave

FlashWeave is a probabilistic graphical modeling tool designed for inferring high-resolution interaction networks from large and heterogeneous microbial sequencing datasets, based on co-occurrence or co-abundance patterns [49]. It is particularly well-suited for microbiome data due to its ability to account for compositional effects characteristic of sequencing data [50], and to handle sparse datasets, explicitly considering zeros to prevent indirect associations (spurious edges) between taxa with similar absence patterns [51]. Additionally, FlashWeave integrates metadata, including environmental factors, as additional nodes in the network, enabling the differentiation of direct microbial associations from indirect ones driven by shared environmental effects [8, 9].

Network calculation was preceded by two pre-processing steps to enhance the reduction of indirect associations, following the approach outlined by Freudenthal et al. [52]. Firstly, the influence of environmental factors on the microbial communities was assessed, namely TOC (%), TN (%), TP (g/kg), CN ratio, pH, and sampling region. Nonmetric multidimensional scaling (NMDS) plots were generated for all data, and the environmental variables were fitted onto the ordinations (refer to the “Statistical analyses,” for details on software and methods). Permutational multivariate analysis of variance (PERMANOVA) was performed to test differences in community composition across regions and edaphic parameters. Statistically significant environmental vectors, scaled by their correlation values, were added to the NMDS plots to visualize their influence on microbial community composition. The analysis identified region as the variable exerting the strongest influence on the communities, and, consequently, network analyses were conducted separately for each region. Secondly, spurious edges caused by rare species were minimized by applying prevalence filters to exclude rare taxa, removing OTUs present in fewer than 10% of the samples in each dataset.

Correlation network construction was performed using FlashWeave (v. 0.19.0) [49] to infer the microbial putative interactions, using the Julia environment (v. 1.7.3) [53] employing the sensitive mode with default settings. To further control for data compositionality, we applied a centered-log-ratio transformation separately to each of the three taxa abundance datasets. The resulting networks were visualized in Cytoscape (v. 3.10.1) [54]. Significant putative interactions between taxa (nodes) were aggregated at the order level to simplify interpretation. Node sizes corresponded to the total number of reads for each order, and node color denoted the number of aggregated genera. Edges represented individual putative interactions, with line thickness denoting the number of interactions between two orders and edge colors indicating positive (blue) or negative (red) correlations. Thus, by integrating region-specific analyses, filtering for rare taxa, and employing a robust network inference tool, our approach minimized spurious putative interactions and generated ecologically meaningful networks that were further validated experimentally.

### HMSC

To further validate the robustness of our findings and confirm predator–prey correlations identified by FlashWeave, we employed Hierarchical Modeling of Species Communities (HMSC), a Bayesian multivariate form of the *Joint Species Distribution Modelling* (JSDM) framework, framed with generalized linear model (GLM) principles [55]. HMSC allows the disentanglement of environmental correlations (shared species responses to environmental predictors) from residual associations (interactions not explained by environmental factors), providing valuable insights into potential biotic interactions [56].

We subjected the data to the HMSC workflow and constructed networks using a hurdle model with the R package Hmsc (v. 3.0–13) [57]. While zero-inflated models are not currently implemented in HMSC, a hurdle model can address this characteristic of sequencing data and consists of two components: one for modeling presence-absence and another for modeling abundance conditional on presence [56]. As both components are statistically independent, the approach enables separate exploration of environmental covariates driving species occurrence versus those influencing species abundance [58]. The first model’s data were truncated to presence-absence (retaining zeros and setting non-zeros to one) and fitted the matrix with a probit (binomial) model. For the second model, zeros were treated as missing values, and the original abundances were analyzed using a normal model. Prevalence filters were applied to exclude rare taxa, removing OTUs present in fewer than 10% of samples (as done with FlashWeave), resulting in a unique dataset for the three phyla.

The fixed explanatory variables included were TOC (%), TN (%), TP (g/kg), pH, and sampling region, with the log-transformed sequencing depth added as a continuous variable to control for variation in sequencing effort [59]. The sampling site and sample code were incorporated as random effects to capture unexplained variation due to spatial structure and unmeasured covariates [56]. To account for the influence of species traits on interspecific interactions, we included functional traits such as nutritional mode (e.g., autotroph, eukaryvore, omnivore) and phylum in the models as input parameters for the estimation of species niches [59]. We built species-to-species association matrices for null models (using sequencing depth only) and full models (including all explanatory variables). Null model matrices reflect habitat-driven and interactive co-occurrence patterns, while full model matrices isolate patterns more indicative of direct interactions by accounting for environmental effects [59]. The models were sampled using Markov chain Monte Carlo (MCMC) algorithms with four parallel chains. Each chain was run for 150,000 iterations, with the first 50,000 iterations discarded as burn-in. The remaining iterations were thinned by retaining every 100th sample, resulting in 1000 posterior samples per chain and 4000 posterior samples in total. Convergence was assessed through the effective sample sizes (ESS) and potential scale reduction factors (PSRF). Model fit was evaluated using predicted values, with explanatory and predictive power quantified as AUC for presence-absence models and *R*^2^ for abundance models.

### Isolation, cultivation, and identification of algae and heterotrophic protists

In order to support the indirect evidence of predator–prey relationships raised by the network analyses, direct evidence was desired. Therefore, potential algal prey and predatory cercozoans were cultured for subsequent food range experiments. Before isolation, small portions of each soil crust (ca. 50 mg) were enriched with Waris-H medium (pH 7) [60]. To establish monoclonal cultures, individual cells were isolated using glass pipettes under an inverted microscope and placed into 24-well plates. Green algae and ochrophytes were cultured in SiO_3_-enriched Waris-H medium. Depending on their required prey, heterotrophs were cultured in wheatgrass medium (WGM) or Waris-H medium, using bacteria or *Saccharomyces cerevisiae* as prey, respectively (generated cultures are available in Tables S9 and S10). The cultures were stored at 15 °C, with a light regime of 14/10 h light/dark, and a light intensity of about 6.5 PPFD (6500-K lamp). All cultures were barcoded by targeting the 18S rRNA-encoding gene.

### Confirmation of predation

Food range experiments were conducted with cercozoan predators and algal prey, according to the availability of cultures generated in this study. Initially, 11 network-indicated putative interactions were qualitatively tested to confirm predation, resulting in the images presented in Fig. 1. Interactions that passed the preliminary testing were subsequently subjected to experiments aimed at quantifying predator feeding rates. Algal cultures not older than 1 month, cultivated in SiO_3_-enriched Waris-H medium, were incubated in the dark a week prior to use. Algal inocula were standardized in their abundance using a Neubauer chamber for both experimental and control sets. The microcosms were set up in triplicate sets in 24-well plates employing Waris-H medium without a nitrogen source, at 15 °C in the darkness to prevent algal growth. Subsequently, approximately 20 cercozoan cells were manually transferred to each well, thus reducing the chance to transfer previous prey of cercozoans. Active predator counts were documented over a period between 20 and 30 days. Chlorophyll autofluorescence intensity served as a proxy for cell density and was measured with a microplate reader (Varioskan Flash, Thermo Fisher Scientific, Waltham, MA, USA). Fluorescence measurements were obtained at 430-nm excitation, 665-nm emission, and a 12-nm bandwidth, using the multipoint setting at 121 reads per well. Fluorescence was recorded at four to six time points, and read averages were calculated. Additionally, fluorescence calibration curves were established for each alga, incorporating increasing algae densities determined with a Neubauer chamber. Algal fluorescence was transformed to algal densities for the final plots.

### Statistical analyses

All statistical analyses were performed using R (v. 4.3.0) [61]. Diversity analyses were calculated with *vegan* (v. 2.6–4) [62], and data manipulation and visualization were achieved using the core *tidyverse* packages (v. 2.0.0) [63], *RColorBrewer* (v. 1.1–3) [64], and *ggrepel* (v. 0.9.5) [65]. Initially, rarefaction curves were calculated with vegan::rarecurve (Figs. S3, S4, S5), demonstrating that all sample replicates reached sufficient saturation. For downstream analysis of the data, the read counts were transformed into relative abundances per sample. Data were then screened to assess differences in community structure between the three regions and to identify outliers. For this purpose, nonmetric multidimensional scaling (NMDS) plots were computed using vegan::metaMDS based on Bray–Curtis dissimilarities (beta diversity), calculated using vegan::vegdist. *p*-values were adjusted for multiple comparisons using the Benjamini and Hochberg method [66]. Two sampling sites (10 samples) were identified as outliers and, consequently, were excluded from further analyses, resulting in a dataset of 23 sampling sites and 116 samples for the 3 regions (Svalbard, *N* = 46; Antarctic Peninsula, *N* = 45; Continental Antarctica, *N* = 20). Edaphic factors, namely TOC (%), TN (%), TP (g/kg), CN ratio, and pH, and the sampling region were incorporated into the NMDS plots using the vegan::envfit function. Biotic factors, specifically the community compositions of Cercozoa, green algae, and ochrophytes, were used as determinants of community structure to evaluate their role as shaping factors and further support the hypothesized predator–prey interactions. For this purpose, principal coordinates analyses (PCoA) were calculated for each data set using vegan::capscale, with community compositions serving as input*.* The PCoAs showed that the first two axes explained 19.3% and 11.2%, 20.7% and 14.25%, and 19.0% and 12.3% of the variation in the cercozoan, green algal, and ochrophyte communities, respectively. The first axis (PCoA1) of each taxon’s community was used to predict those of the others. To assess differences in beta diversity across sampling regions and edaphic factors, we applied permutational multivariate analyses of variance (PERMANOVA) with vegan::adonis2 using 999 permutations.

Venn diagrams were generated with *ggvenn* (v. 0.1.10) [67], and the final figures were plotted to approximately depict OTU abundances using *eulerr* (online v. 6.1.1) [68]. Alpha-diversity metrics were calculated with *vegan*, and statistical comparisons were conducted using ANOVA and Tukey HSD post hoc tests, with the packages *ggpubr* (v. 4.3.1) [69] and *rstatix* (v. 4.3.1) [70].

Analyses of covariance (ANCOVA) were employed on the data generated with the feeding rate experiments, to assess whether changes in algae densities and predator counts were influenced by time, experimental conditions, or their interaction. The assumptions of homogeneity of regression slopes, homogeneity of variances, and normality of residuals were assessed. *p*-values underwent multiple-testing correction using the Benjamini and Hochberg (1995) method [66]. Visualization was conducted using *scales* (v. 1.2.1) [71] in addition to the core *tidyverse* packages (v. 2.0.0) [63]*.*

## Results

### Sequencing results

Our first aim was the production of multiple, independent metabarcoding datasets of a predator group (Cercozoa) and their respective putative prey (here green algae and Ochrophyta). Applying the metabarcoding methodology established by Fiore-Donno et al. [39], we generated 10.0 million paired reads for Cercozoa. Furthermore, using our newly developed metabarcoding protocols for green algae (Archaeplastida) and ochrophytes (Heterokontophyta), we produced 12.4 million and 10.7 million paired reads, respectively. The specificity of the respective sequencing approaches for Cercozoa and green algae were high with 96.1% and 80.6% of the sequences representing respective target taxa. For ochrophytes, only 28.7% stemmed from the targeted taxa, diatoms and chrysophytes, in nearly equal proportions. Despite considerable nontarget amplification in the Ochrophyta dataset, saturation was reached in all datasets, for all sites and replicates (Figs. S3, S4, S5), rendering all datasets suitable for further analyses. In total, 604 cercozoan, 191 green algal, and 80 ochrophyte unique OTUs were generated across 116 samples. On average, each sample contained 76 cercozoan OTUs (range: 13–164, ± 3.1), 40 green algal OTUs (15–81, ± 1.4), and 11 Ochrophyta OTUs (2–33, ± 0.6, detailed OTU counts and taxonomic affiliations are available in Tables S1, S2, S3).

### Community composition

To illustrate whether our newly developed protocols produced distinguishable and representative results for the community composition and local diversity of the studied regions, we conducted alpha-diversity analyses for each dataset and performed interregional comparisons.

**Fig. 2:**
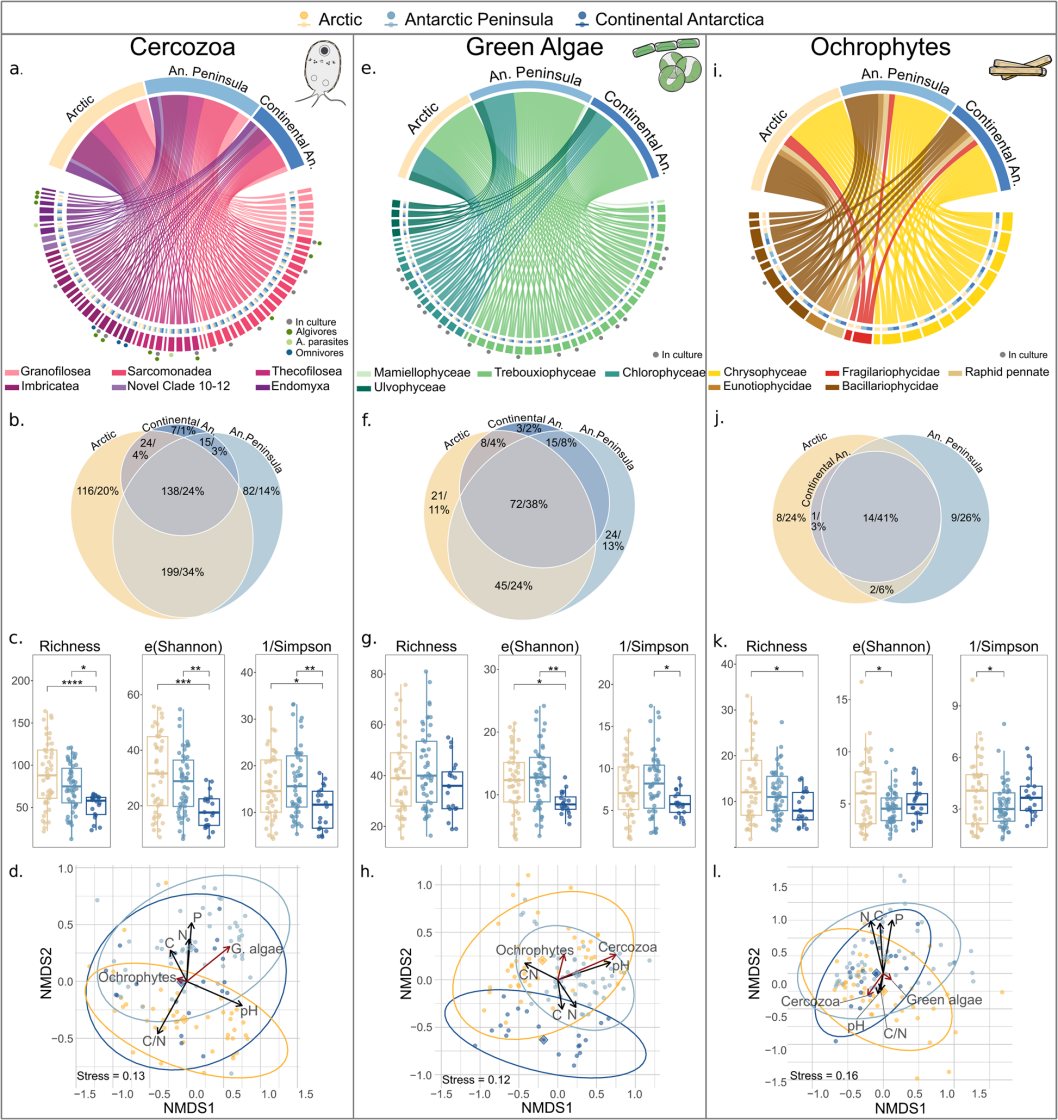
Diversity measures of Cercozoa, green algae, and ochrophytesacross three Polar regions. Chord plots (**a**, **e**, and **i**) depict genus richness identified for all regions within the targeted taxa. The chords connect the regions (yellow and blue shades) with the corresponding genera, while different chord colors represent distinct classes. Circles next to the taxon names indicate cultivated taxa (gray), specifically highlighting algivores (dark green), and algae parasites (light green) in Cercozoa. Chord plots in Supplementary Figs. S6, S7, and S8 display genera names. Venn diagrams (**b**, **f**, and **j**) represent unique and shared OTUs within and between sampling regions. Globe sizes are approximately proportional to their abundance. Box plots (**c**, **g**, and **k**) display three *α*-diversity indices: (1) OTU richness, (2) e(Shannon) for exponential Shannon, and (3) inverse Simpson. Significance codes indicate differences between means calculated by ANOVA and subsequent Tukey-HSD test and are denoted as follows: *p < 0.05, **p < 0.01, ***p < 0.001, and ****p < 0.0001. Nonmetric multidimensional scaling ordinations (NMDS, **d**, **h**, and **l**) based on Bray–Curtis dissimilarities for the three taxa. The structures (PCoA1) of each of the three communities, used as predictors for the other two, are indicated by red arrows. For instance, the structures of green algae and ochrophytes were used to predict the structure of Cercozoa, and similar predictive analyses were conducted for green algae and ochrophytes. Region centroids are indicated by diamonds and follow the same color coding as the legend. The confidence ellipses were drawn at the 0.95 level, indicating the regions within which 95% of the data points are expected to lie. The influence of abiotic variables (pH, C, N, P, and C/N ratio) with the highest correlation to the ordination axes are indicated by black arrows. Scaling was performed using *k* = 3 dimensions; only the two first dimensions are visualized

The cercozoan richness was dominated by Sarcomonadea, with 70% of the total 604 OTU count (39 genera). Imbricatea and Thecofilosea contributed 11% (26 genera), and 9% (17 genera), respectively (Fig. 2a; Supplementary Fig. S7 displays genera names). Numerous OTUs in all datasets were identified only at broader taxonomic levels, a common limitation stemming from incomplete reference databases. Among 352 Cercozoa OTUs that could not be identified up to genus level, 65% were assigned equally to the families Allapsidae and Sandonidae (both Sarcomonadea). Twenty-four percent of OTUs, mostly representing Sarcomonadea, were present across all three regions (Fig. 2b). Alpha-diversity metrics indicated that Svalbard exhibited the highest cercozoan diversity across the three metrics, namely OTU richness (*F* = 10.368, *p* = 7.34e-5), exponential Shannon (*F* = 8.809, *p* = 0.000278), and inverse Simpson (*F* = 4.955, *p* = 0.009; Fig. 2c; Table S11). Thus, these results demonstrate that Svalbard exhibits higher species richness and a more evenly distributed community than the two Antarctic regions.

Green algae were especially rich in Trebouxiophyceae, representing 71% of the total 191 OTUs across 28 genera. Chlorophyceae contributed 24% of OTUs, whereas Ulvophyceae and Mamiellophyceae accounted for seven and one OTUs, respectively (Fig. 2e, Supplementary Fig. S7). Only 37% of OTUs appeared in all three regions, and the most widespread genera belonged to Trebouxiophyceae and Ulvophyceae (Fig. 2f). Most of the unique genera (eight) were observed in both Antarctic regions, while two green algal genera appeared exclusively in Svalbard. Moreover, 17% of OTUs were identified until class or order levels. The OTU richness did not vary among regions (*F* = 1.853, *p* = 0.162). Contrastingly, both the inverse Simpson (*F* = 3.88, *p* = 0.023; Fig. 2g; Table S11) and exponential Shannon metrics (*F* = 3.88, *p* = 0.003), supported by subsequent Tukey tests (*p* = 0.017 and *p* = 0.0017), revealed differences between the two Antarctic regions. Additionally, results for exponential Shannon illustrated a higher diversity in Svalbard than in Continental Antarctica (Tukey test, *p* = 0.0191). Thus, these findings reveal a consistent OTU richness across regions but indicate a more uneven community distribution in Continental Antarctica compared to Svalbard and the Antarctic Peninsula.

The ochrophyte sequencing yielded 80 OTUs, with the class Bacillariophyceae dominating the diatom relative abundance. The latter encompassed 34 OTUs across 12 genera (Fig. 2i, Supplementary Fig. S8). Forty-one percent of all diatom OTUs within seven genera were common in all regions, whereas 26% and 23% of OTUs were unique to the Antarctic Peninsula and Svalbard, respectively (Fig. 2j). Nevertheless, most genera occurred widespread, with only three being unique to Svalbard and one to the Antarctic Peninsula. Nine OTUs were classified under the taxonomically uncertain operational categoric name “raphid pennates,” corresponding to Bacillariophyceae. Of the latter, five were found in all regions, while four were unique to the Antarctic Peninsula. Among the 41 chrysophyte OTUs, half were assigned to 5 genera, dominated by *Spumella* (50%) and *Ochromonas* (25%). The remaining 21 taxa were only assigned to clades. While most chrysophyte clades and genera appeared across the three regions, *Paraphysomonas* was only found in Svalbard. Svalbard’s Ochrophyta OTU richness was higher than in Continental Antarctica (Tukey test, *p* = 0.011). Additionally, both exponential Shannon and inverse Simpson demonstrated differences between Svalbard and the Antarctic Peninsula (Tukey tests, *p* = 0.0191 and *p* = 0.037, respectively; ANOVA results in Table S11). Taken together, these findings demonstrate higher species richness and evenness in Svalbard for the studied ochrophytes.

We conclude that the three different metabarcoding approaches successfully exhausted the present diversity and are thus suitable for further analyses.

### Biotic and abiotic factors shape microbial communities

To investigate whether the hypothesized predator–prey interactions represent a shaping factor for these microbial communities, beta-diversity analyses were performed with emphasis on biological factors as determinants (Fig. 2d, h, l). Our analyses indicated the presence of a biotic impact of Cercozoa on green algae (refer to Table S12 for PERMANOVA results). The cercozoan community composition explained 5.4% of the green algal community composition (*R*^2^ = 0.0537, *p* < 0.001). Vice versa, the green algal community composition explained some variation in Cercozoa, albeit to a lower extent (3.5%; *R*^2^ = 0.0348, *p* < 0.001). The ochrophyte community structure explained 2.1% and 3.0% (*R*^2^ = 0.0206, *p* < 0.001; *R*^2^ = 0.0307, *p* < 0.001) of the cercozoan and green algal community composition’s variation, respectively. Finally, Cercozoa and green algae had only minor structuring effects on ochrophytes, accounting for 2.2% and 1.7% of their variation (*R*^2^ = 0. 0224, *p* < 0.001; *R*^2^ = 0. 0.0169, *p* < 0.001), respectively.

To compare the extent to which biological variables affect the community composition of the investigated taxa with the impact of abiotic factors, we also quantified to which extent the respective community compositions are affected by abiotic variables. The regional factor had the strongest effect on the cercozoan (*R*^2^ = 0.1208, *p* < 0.001) and green algal communities (*R*^2^ = 0.1429, *p* < 0.001), explaining 12.1% and 14.3% of the variation, respectively. While for the ochrophyte community composition region was the most influential factor (*R*^2^ = 0.0571, *p* < 0.001), its effect was comparatively lower, with only 5.7% of explained variation. All three taxa were highly influenced by the pH, explaining the variation in the communities of Cercozoa (10.6%), green algae (8.2%), and ochrophytes (4.7%). In contrast, TOC (%), TN (%), TP (g/kg), and the CN ratio contributed to a lower extent, explaining marginal percentages (< 3.7%) of the variation in the three ordinations.

### *Determination of putative predator–prey interactions *via* cross-kingdom co-occurrence networks*

To shift from a community perspective to specific putative interactions between Cercozoa and their prey, we employed co-occurrence network analyses as an estimation for a microbial food web.

**Fig. 3:**
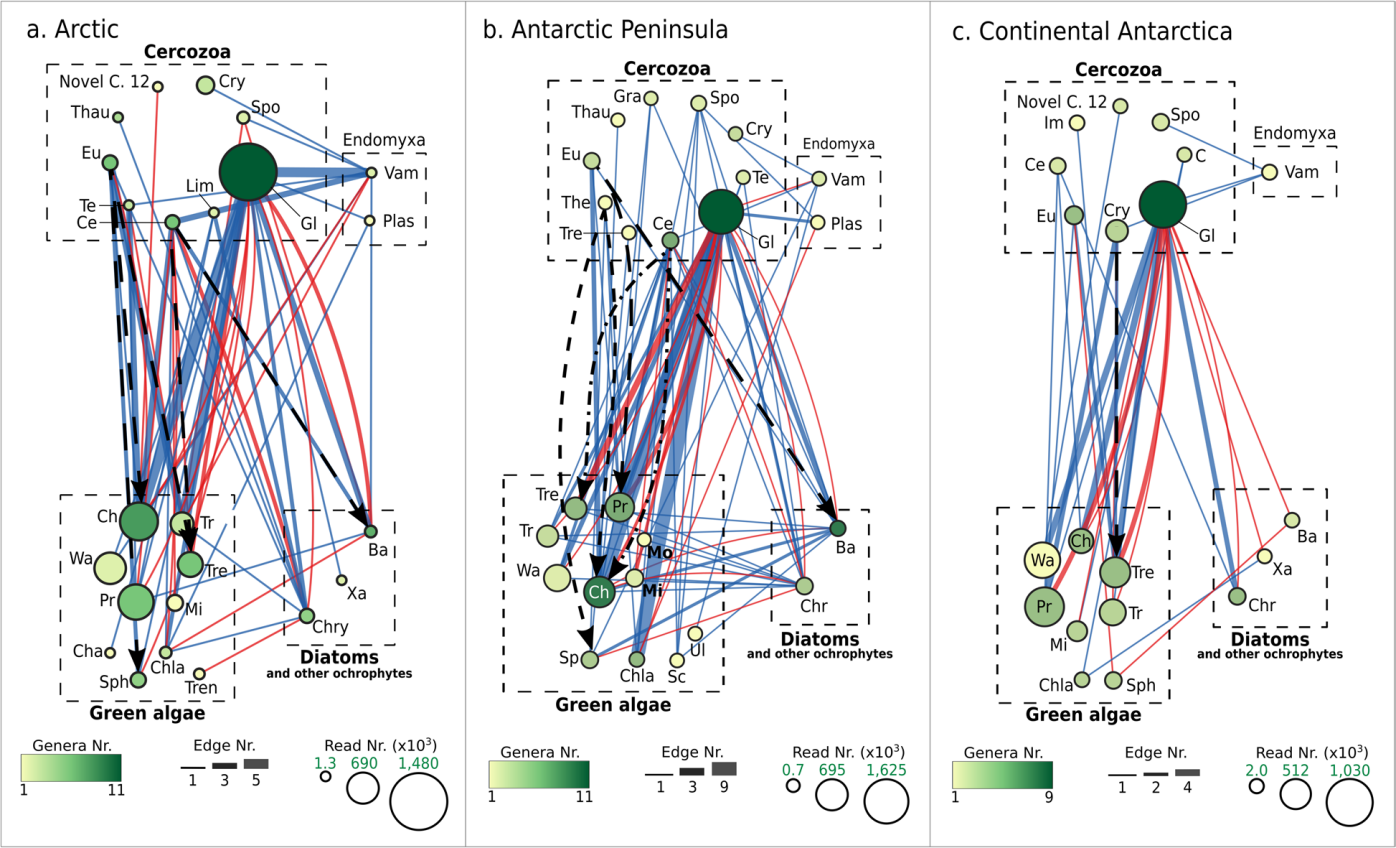
Cross-kingdomco-occurrence networks of Cercozoa, green algae, and ochrophytes across three Polar regions, as an indicator for putative food web interactions. Significant positive and negative putative interactions detected between taxa are depicted for the three studied regions. Nodes represent genera grouped at order level; node size is proportional to the total number of reads for each order, and node color indicates the number of genera aggregated. Edges represent putative interactions between taxa (blue lines — positive putative interactions; red lines — negative putative interactions). Edge thickness represents the number of individual aggregated edges. Black dashed arrows indicate experimentally tested putative predator–prey relationships. Abbreviations: Novel C. 12, novel clade 12; Gra, Granofilosea X; Thau, Thaumatomonadida; Eu, Euglyphida; The, Thecofilosea X; Tre, Tremulida; Te, Tectofilosida; Ce, Cercomonadida; Lim, Limnofilida; Gl, Glissomonadida; Spo, Spongomonadida; Cry, Cryomonadida; C, Cercozoa X; Vam, Vampyrellida; Plas, Plasmodiophorida; Ch, Chlorellales; Wa, Watanabeales; Tre, Trentepohliales; Tr, Trebouxiophyceae X; Pr, Prasiolales; Cha, Chaetophorales; Sph, Sphaeropleales; Chla, Chlamydomonadales; Mi, Microthamniales; Tre, Trebouxiales; Sc, Scotinosphaerales; Ul, Ulotrichales; Mo, Monomastigales; Ba, Bacillariophyceae; Xa, Xanthophyceae; Chry, Chrysophyceae

With FlashWeave, on the OTU level, 407 putative interactions (edges, Fig. 3) were identified among 306 taxa in Svalbard, 422 among 303 taxa in the Antarctic Peninsula, and 140 among 135 taxa in Continental Antarctica. On class level, this equals 30, 28, and 23 nodes for Svalbard, the Antarctic Peninsula, and Continental Antarctica, respectively. The corresponding class-level-aggregated putative interaction counts were 157, 153, and 71 for Svalbard, the Antarctic Peninsula, and Continental Antarctica, respectively. These findings align with the observed lower richness of Cercozoa and ochrophytes in Continental Antarctica.

Network topological features were computed for the OTU level and the aggregated order level (Table S13). Here, the calculations for the latter are described, as taxonomic orders resemble approximately functional groups with few exceptions (e.g., the class Glissomonadida accommodates almost exclusively bacterivores, except for the algivorous family Viridiraptoridae). The connectivity of the networks, as indicated by the average edge degree, was lower for Continental Antarctica (approximately six putative interactions) compared to Svalbard and the Antarctic Peninsula (10–11 putative interactions). The Antarctic Peninsula exhibited the highest average clustering coefficient and network density, and the shorter average path lengths, whereas Continental Antarctica displayed the opposite trend. Taken together, these results demonstrate that the Antarctic Peninsula harbors a more tightly connected, denser, and potentially more stable network compared to the other regions.

Intra-domain putative interactions predominated across the three regions, comprising 310 (78.1%), 290 (68.7%), and 89 (63.6%) in Svalbard, the Antarctic Peninsula, and Continental Antarctica, respectively. Inter-domain putative interactions predominantly involved cercozoan taxa in all regions. Specifically, Cercozoa exhibited 72 (17.7%) putative interactions at the OTU level with algae in Svalbard. However, only 26, i.e., 6.4% of the respective interactions, included algivorous Cercozoa and thus represented putative predator–prey interactions. In the Antarctic Peninsula, 20 interactions (4.7% of 422) corresponded to putative predator–prey interactions, while in Continental Antarctica there were 13 interactions (9.3% of 140) of such. Notably, the Glissomonadida occupied at least half of the total putative interactions in each region, despite the vast majority of Glissomonadida species do not prey on algae. Interestingly, few putative interactions were found between green algae and ochrophytes across all three regions, particularly in Continental Antarctica. Taken together, these findings suggest that Cercozoa play a crucial role in structuring microalgal communities. However, predation may not be the sole interaction explaining the found correlations.

To further validate the results of FlashWeave and provide an additional layer of insight into potential predator–prey interactions, we employed HMSC to disentangle the environmental and biotic drivers shaping the networks. Using the HMSC hurdle model approach, we constructed co-occurrence and abundance networks for null and full models to identify residual correlations that are likely indicative of direct interactions (Supplementary 10, Fig. S9). HMSC yielded species-to-species association matrices for presence-absence and abundance data, incorporating environmental variables and functional traits to account for both ecological processes and shared habitat use. Null models, which include only sequencing depth as a covariate, captured a combination of co-occurrence patterns from shared environmental preferences and interactive effects. Full models, which include additional environmental predictors (i.e., TOC (%), TN (%), TP (g/kg), pH, and region), refined these associations by isolating residual correlations. Notably, the HMSC networks identified a similar proportion of predator–prey interactions to FlashWeave, namely 7.5% and 4.8% (Supplementary Table S16), for co-occurrence and abundance, respectively.

### Effect verification and size estimation of putative predator–prey interactions

In order to provide direct evidence of the putative predator–prey interactions identified in the network analyses, feeding range experiments were conducted with algivorous predators and their putative algal prey. The FlashWeave correlation network guided our selection of correlations for experimental validation and served as the basis for hypothesizing predator–prey interactions. Additionally, we used the HMSC models to inform these interactions by accounting for environmental covariates, species traits, and hierarchical dependencies, thereby refining the ecological context of the putative associations.

For this, we isolated and barcoded potentially algivorous taxa and their respective prey. According to the sequencing results, algivorous *Cercomonas*, *Rhogostoma*, *Fisculla*, *Assulina*, and *Euglypha* were common in all study regions. Moreover, *Rhizaspis*, *Viridiraptor*, and *Eocercomonas* appeared in both Svalbard and the Antarctic Peninsula but were missing in Continental Antarctica. Among algivorous Endomyxa, *Vampyrella* and *Thalassomyxa* (Endomyxa) were detected exclusively in Svalbard, while *Leptophrys* was detected in the two Antarctic regions. We successfully established cultures of algivorous *Cercomonas*, *Rhogostoma*, *Fisculla*, and *Euglypha* and subjected them to the experiments.

The isolation and culturing of green algae for subsequent testing resulted in 38 cultures of barcoded green algae. Isolated algae belonged to the classes Chlorophyceae and Trebouxiophyceae, while no representatives from Mamiellophyceae or Ulvophyceae were observed or cultured. Most cultures were identified to genus or species level, except for eight, which were assigned only to the orders or families Chlamydomonadales, Chlorellales, Chlorellaceae, Radiococcaceae, and Haematococcaceae. Five ochrophyte cultures were established from Arctic isolates. These included three *Pinnularia* sp. isolates originating from different sites, and one *Nitzschia perminuta*, all of which were present in the environmental sequencing results.

**Fig. 4:**
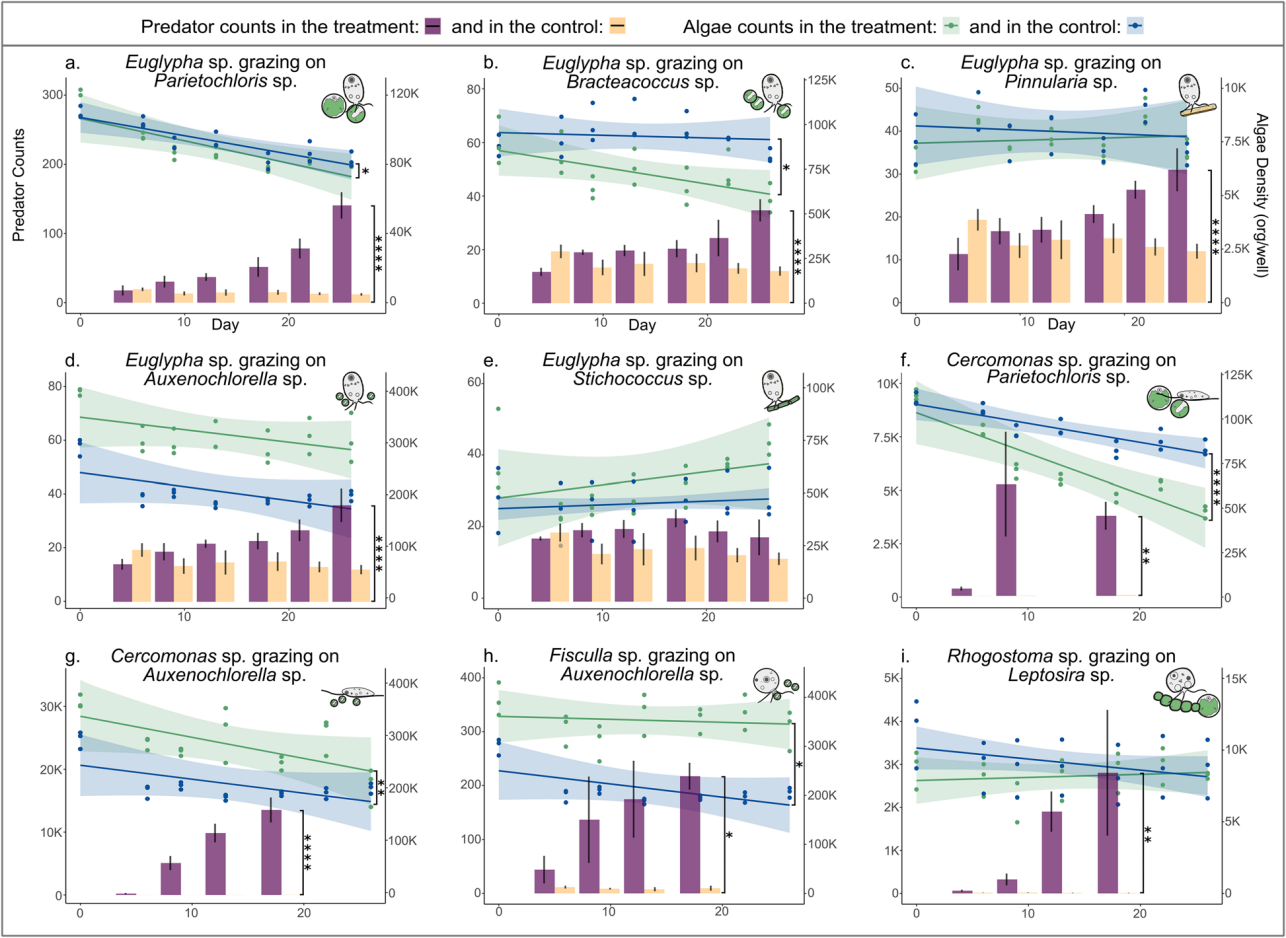
Experimental validation of putative predator–prey interactions. Yellow bars represent predator counts for control conditions, while purple bars depict their counts in co-culture with algal prey (left *y*-axis). Blue lines represent algae counts in control conditions, while green lines indicate algae counts under predation pressure (right *y*-axis). Error bars accompanying each bar display standard deviations. Significance codes derived from ANCOVA analyses indicate differences between the treatment and control slopes for both predator counts (Table S14) and algae fluorescence (Table S15). The codes are denoted as follows: *p < 0.05, **p < 0.01, ***p < 0.001, and ****p < 0.0001

The cultures generated in this study enabled the testing of 11 network-indicated putative predator–prey relationships (Fig. 4). Initially, all putative interactions underwent qualitative testing to confirm predation. Notably, two putative predator–prey interactions could not be confirmed, namely, the interactions involving the highly motile alga *Chloromonas* with the cercozoan *Cercomonas* and *Fisculla* were not validated. Subsequently, to move from qualitative evidence to quantitative results, feeding rate experiments were conducted for the remaining nine verified predator–prey interactions. ANCOVA analyses of predator count slopes demonstrated considerable growth in eight of the tested putative interactions, except for *Euglypha* feeding on *Stichococcus* (Fig. 4e, Table S14). Nevertheless, in all tested interactions, the predators exhibited food vacuoles containing the respective algae at various stages of digestion (Fig. 1). Moreover, a decrease in algal abundance slopes was detected in five putative interactions (Table S15), indicating that while predation was observed and statistically significant in most tested putative interactions, it had a negligible impact on the overall abundance of certain algal populations in this experimental setup.

**Fig. 5:**
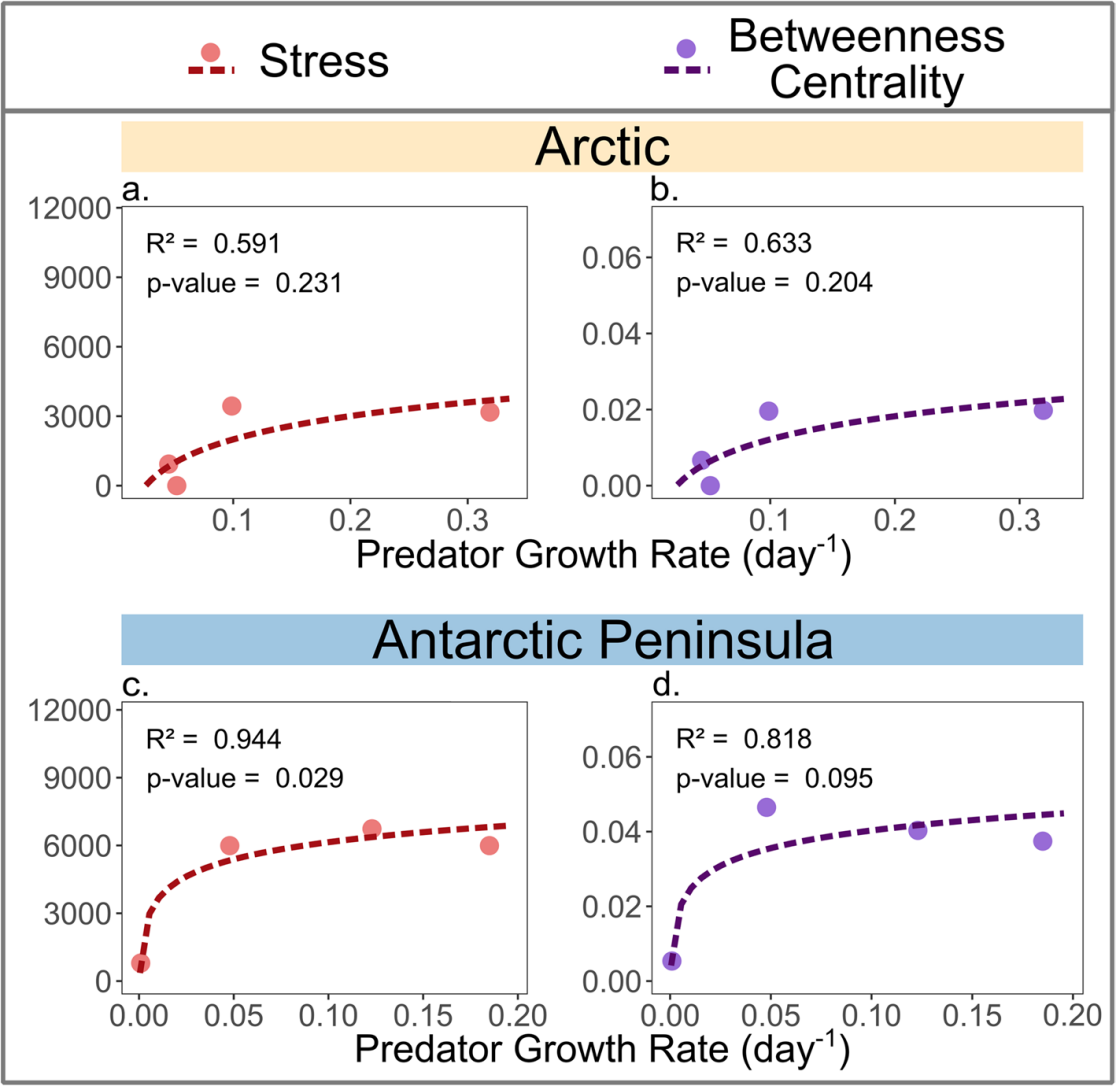
Correlations between two network topological features — (1) stress (**a** and **c**) and (2) betweenness centrality (**c** and **d**) — and predator growth rate. Each topological feature is represented by its calculated value (*y*-axes), while predator growth rates were calculated as the exponential increase of predator populations per day (*x*-axes)

We explored various correlations between network topological features and the calculated predator growth rate of each verified predator–prey interaction, aiming to associate our experimental quantitative data with the network analysis derived from network processing. Two key node topological features, stress and betweenness centrality, were found to increase with higher predation growth rates (Fig. 5). Both topological features are centrality measures that highlight the critical roles of certain taxa within a network. Stress centrality quantifies a node’s importance based on the number of times it acts as a bridge along the shortest paths of other nodes [72], while betweenness centrality indicates a node’s influence on the interactions between other nodes in the network [73]. Thus, these findings imply a potential relationship between predation growth rate and the importance of predators in the food web, suggesting that more effective predators are likely to be more central and play critical roles in maintaining the network’s stability and functionality.

## Discussion

Inferring microbial interactions involves establishing hypothesized relationships based on the association patterns of taxa in environmental sequencing data and ultimately experimentally validating genuine ecological interactions [1, 34]. Using this approach, cross-kingdom network inference allowed us to predict predator–prey relationships between cercozoans and their putative prey: green algae and ochrophytes. By combining DNA-based amplicon sequencing datasets for Cercozoa and their putative prey, we produced comprehensive databases, suitable for cross-kingdom network analyses. Trait assignment revealed that the majority of network-indicated associations were unlikely to represent predator–prey interactions. However, from the tested possible predator–prey interactions, 82% of network-indicated predator–prey interactions could be validated.

### Estimation of biotic interactions

Predation is a major driving force within microbial communities, which directly influences population dynamics, community composition, and microbial resistance [74–76]. Given predation’s ecological significance, we aimed to investigate the robustness of inferring predator–prey interactions from correlation data by experimental validation. As a first step, we estimated the impacts of biotic interactions by (1) determining the extent to which the predator and putative prey communities shape each other and (2) inferring microbial interactions through network analyses. Analysis of beta diversity revealed that biotic factors had a key role in shaping microbial communities, closely following the dominant influences of region and soil pH. Particularly, the interactions between cercozoans and green algae were important. The community composition of green algae exerted a 1.5 times larger effect on the Cercozoa than vice versa, suggesting a notable impact of cercozoan predation on green algae. The structuring effect of ochrophytes on the green algal community was approximately 1.8-fold stronger than the reverse. Moreover, ochrophytes and cercozoans had a similar and small impact on each other’s community composition. Notably, the shaping effect of cercozoans on green algae was over twice as strong as their effect on ochrophytes. Therefore, we hypothesized that network analyses would estimate fewer putative predator–prey interactions between cercozoans and ochrophytes. These findings highlight the shaping role of biotic interactions over microbial assemblages, consistent with previous research that emphasizes the importance of trophic and other biotic interactions, along with environmental effects, in structuring microbial communities [77, 78], and the complex and diverse nature of the associations [2, 79].

As microbial interactions influence population dynamics, microbial survey datasets are expected to reveal detectable signatures of these interactions [2]. Our cross-kingdom network analysis of polar biocrusts, known for their low complexity and reduced biodiversity, allowed us to predict, and subsequently test, predator–prey associations between cercozoans, green algae, and ochrophytes. We found that the majority of the correlations calculated for Svalbard, the Antarctic Peninsula, and Continental Antarctica were positive inter-domain putative interactions, accounting for 81%, 79%, and 76% of the total correlations in each location, respectively. Interestingly, this trend extended to putative predator–prey interactions, with the majority also being positive. The former results imply that when assessing network-generated putative predator–prey interactions, attention should be given to all correlations, regardless of their sign (positive or negative). While predators and prey are expected to positively correlate over some spatial scales that allow predators to maximize prey encounters, at smaller scales, effective predators are expected to reduce or eliminate prey populations [77]. Moreover, relationships can be time-lagged [1] as a result of predation and environmental or population changes. Thus, the pervasive occurrence of positive putative interactions across all three regions, particularly among potential predator–prey interactions, implies that the nature of putative predator–prey interactions cannot be directly attributable to any predefined biological functions.

Only 72 (17.7%), 86 (20.4%), and 46 (32.9%) of the total correlations occurred between predatory protists and green algae or ochrophytes in Svalbard, the Antarctic Peninsula, and Continental Antarctica, respectively. However, considering that only a minor fraction of Cercozoa involved in these correlations are algivores, trait assignment revealed that 6.4%, 4.7%, and 9.3% of the total correlations link predators to suitable prey for each respective region in FlashWeave networks, which was further supported by similar numbers in HMSC networks, showcasing the robustness of our approach. Associations with taxa within Glissomonadida accounted for at least half of the total putative interactions in each region, despite the order comprising almost exclusively bacterivores [33]. Accordingly, it probably represents a hub taxon within these ecosystems. Glissomonads are fast-growing microorganisms that might thrive during periods of favorable conditions and become abundant in the soil matrix, explaining their high abundance. The association of glissomonads and algae, if an interaction exists, could be driven by shared environmental preferences or indirect effects mediated through the microbial community rather than direct predation. Another possibility is that glissomonads might be attracted towards microalgae, which are among the dominant carbon fixers in polar areas where plant life is sparse or absent [17], and release exudated carbon compounds into the environment [80]. Glissomonadida might either scavenge those released carbon compounds as osmotrophs [33, 81] or prey upon algae-associated carbon-scavenging bacteria, a process rather similar to the microbial loop relying on plants [82].

### Confirmation of biotic interactions

To provide a basis for comparison, we aimed to generate three replicated networks from comparable but sufficiently different communities. Among the three regions, species richness and evenness for Svalbard and the Antarctic Peninsula were consistently higher, especially for cercozoans and green algae (Fig. 2), contrasting with Continental Antarctica. Despite this, all regions shared over half of their OTUs, while specialist OTUs comprising between 1 and 26%, indicating a considerable overlap in species composition (likely due to the presence of cosmopolitan or bipolar species), and some degree of endemicity. Moreover, our results were in line with previous surveys which concluded that the Antarctic Peninsula accommodates a higher protistan biodiversity compared to the arid and more extreme continental regions, such as the Thala Hills, where our samples originated from [21, 83–85]. Beta-diversity analyses supported the presence of different microbial assemblages across the three regions and found this parameter as the primary factor driving variation. Association with soil abiotic factors indicated low and varied effects on community structure, with pH showing the strongest correlation. Thus, these findings collectively suggest that local climate, followed by soil parameters, play significant roles in shaping soil protist communities, in line with past studies [6, 86]. We conclude that the community composition, although being similar, indeed varied significantly across the three sampled regions, enabling us to produce the expected distinct, replicated networks of similar communities, as evidenced by the comparable numbers of calculated and confirmed putative predator–prey interactions. Moreover, by focusing on low-diverse environments and predator–prey associations, we aimed and successfully sampled the three communities exhaustively, generating high saturation in our sequencing data.

To experimentally confirm the predatory impact of cercozoans, we complemented network-analyses with a trait-based approach. With co-culture experiments, we quantitatively measured the impact of protistan predation pressure on both the algal prey and the predator, showing substantial effects of predation in most interactions, evidenced by a significant increase of the mean counts of the predators in nine of 11 experiments. Only two interactions, involving the highly motile alga *Chloromonas*, were not validated. Thus, 82% of the associations were confirmed, and coupling network analyses with a trait-based approach was effective in capturing important predator–prey dynamics in the studied biocrusts.

The growth rate experiments further revealed that *Cercomonas* sp., *Fisculla* sp., and *Rhogostoma* sp. were the most vicious predators, i.e., the predators with higher growth rates in co-culture with their prey. Here, it must be addressed that the laboratory experiments do not mirror natural conditions: the experiments were conducted under exclusion of competitors and under optimized conditions, i.e., high prey densities, nutrient limitation, and in liquid media. We chose to do so, since the opaque nature of soil hampers direct observation and thus the validation and enumeration of biotic interactions. It remains to be seen whether with technical progress direct measurements from the environment can supplement indirect, correlational measurements as reported in this paper.

Nonetheless, we report a correlation of predator network statistics, increasing stress and betweenness centrality, in two independent networks. Accordingly, it seems important predators can be predicted from their network statistics (Fig. 4). In the third network derived from Continental Antarctica, the same general concept might apply; however, due to the absence of many of the sampled predator–prey combinations, this could not be verified.

## Conclusions

Our results underscore the utility of network analyses for inferring predator–prey interactions while also emphasizing the importance of integrating these analyses with trait-based approaches to enhance prediction accuracy. This combined methodology increases confidence in identifying true biological interactions, demonstrating the effectiveness of a systematic approach for studying ecological networks. Further studies applying the same trait-based approach and focusing on bacteria-protist interactions are key to deepen our understanding of microbial networks where protists exert a key predatory role. Our developed methodology is largely similar to methods used in researching bacterial and protistan interactions, i.e., the involvement of separate metabarcoding datasets and interkingdom network calculations. We are confident that the here presented workflow facilitates the identification of vicious protistan predators of bacteria.

## Supplementary Information

Supplementary figures: Figure S1. In silico comparison of amplification efficiency of diatom designed and literature primers. All reactions were conducted with the forward primer EukF1. a. Total matched accessions of every tested PCR. The non-redundant SILVA database had 1,571 sequences within the Bacillariophyta at the time of the study. b. Major terrestrial taxa of the three diatom classes are presented. Results are based on a perfect match (0 mismatches) on the SILVA database. Figure S2. In silico comparison of amplification efficiency of green algae primers. All reactions were conducted with the forward primer EukF1. a. Total matched accessions of every tested PCR. The non-redundant SILVA database had 1,908 Chlorophyta and 4,738 Charophyta sequences at the time of the study. The compared methodology [ 27 ] comprise general eukaryotic primers, which expectedly matched a large number of eukaryotic accessions. b. Major terrestrial microalgae taxa are shown within each phylum. Total Embryophyta matched accessions are also shown. Since the developed protocol was intended for microalgae analysis, we aimed to develop specific primers to avoid the amplification embryophytes. Results are based on a perfect match (0 mismatches) on the SILVA database. Figure S3. Rarefaction curves per sampling sites, Cercozoa. Figure S4. Rarefaction curves per sampling sites, green algae. Figure S5. Rarefaction curves per sampling sites, ochrophytes. Figure S6. Cercozoan genera by sampling regions. Figure S7. Green algal genera by sampling regions. Figure S8. Ochrophyte genera by sampling regions. Figure S9. HMSC species-to-species co-occurrence and correlation networks for Cercozoan algivores and bacterivores with microalgae, inferred using presence-absence and abundance models. Panels (a–d) depict associations between cercozoan eukaryvores and omnivores with green algae and ochrophytes, while panels (e–h) show associations between cercozoan bacterivores and microalgae. The presence-absence models (a, b, e, f) were inferred using a probit model, whereas the abundance models (c, d, g, h) were based on a normal model. Each model includes both a null version (a, c, e, g) to represent random patterns and a full version (b, d, f, h) that incorporates environmental covariates. The correlations marked with black squares represent interactions validated through laboratory testing.

Supplementary Tables: Supplementary Table S1: Count data Cercozoa. Table S2: Count data Green Algae. Table S3: Count data Ochrophytes. Table S4. Sampling sites. Table S5. Diatom primers and tags designed for this study*. Table S6. Green algae primers and tags designed for this study*. Table S7. Primers used in this study. Table S8. Cercozoa, green algae, and ochrophytes included in the internal standards of each run. Table S9. Cercozoa cultures established in this study. Table S10. Algae and ochrophyte cultures established in this study. Table S11. One-way ANOVA comparisons of alpha diversity indices — OTU richness, exponential Shannon, and inverse Simpson— across polar biocrusts samples (*N* = 116) from Svalbard, the Antarctic Peninsula, and Continental Antarctica. Results presented separately for Cercozoa, green algae, and ochrophytes. The table displays F-values with degrees of freedom (df) for the nominator and denominator, along with associated p-values derived from one-way ANOVA comparisons. Table S12. PERMANOVA results for taxonomic groups across environmental factors. Analysis of polar biocrusts samples (*N* = 116) from Svalbard, the Antarctic Peninsula, and Continental Antarctica. Table S13. Network topological features, including inter- and intra-domain co-occurrences for Cercozoa, green algae, and ochrophytes. N = 116. Table S14. Summary of the ANCOVA models for predator counts. Table S15. Summary of the ANCOVA models for algae counts. Table S16. Summary of co-occurrence and correlation interactions identified in Cercozoan-algae HMSC networks across models.

## Data Availability

The analyzed sequencing data was submitted to NCBI GeneBank SRX25611038, SRX25611037, and SRX25611036 and are available under the following link: https://www.ncbi.nlm.nih.gov/bioproject/PRJNA1144814 All scripts to reproduce the work presented in this paper are available in GitHub under the following link: https://github.com/CrstnM/EnhancingMicrobialPredator-PreyDetection_MartinezRendon_etal. EnhancingMicrobialPredator-PreyDetection_MartinezRendon_etal.

## Abbreviations

rRNA: Ribosomal RNA
OTUs: Operational taxonomic units
