# Supplementary material for: Enhancing microbial predator–prey detection with network and trait-based analyses
Source: Microbiome. 2025 Feb 4;13:37. doi: 10.1186/s40168-025-02035-8 (PMC11792678; doi:10.1186/s40168-025-02035-8)
Supplement: Supplementary file 2 — Supplementary figures: Figure S1. In silico comparison of amplification efficiency of diatom designed and literature primers. All reactions were conducted with the forward primer EukF1. a. Total matched accessions of every tested PCR. The non-redundant SILVA database had 1,571 sequences within the Bacillariophyta at the time of the study. b. Major terrestrial taxa of the three diatom classes are presented. Results are based on a perfect match (0 mismatches) on the SILVA database. Figure S2. In silico comparison of amplification efficiency of green algae primers. All reactions were conducted with the forward primer EukF1. a. Total matched accessions of every tested PCR. The non-redundant SILVA database had 1,908 Chlorophyta and 4,738 Charophyta sequences at the time of the study. The compared methodology [ 27 ] comprise general eukaryotic primers, which expectedly matched a large number of eukaryotic accessions. b. Major terrestrial microalgae taxa are shown within each phylum. Total Embryophyta matched accessions are also shown. Since the developed protocol was intended for microalgae analysis, we aimed to develop specific primers to avoid the amplification embryophytes. Results are based on a perfect match (0 mismatches) on the SILVA database. Figure S3. Rarefaction curves per sampling sites, Cercozoa. Figure S4. Rarefaction curves per sampling sites, green algae. Figure S5. Rarefaction curves per sampling sites, ochrophytes. Figure S6. Cercozoan genera by sampling regions. Figure S7. Green algal genera by sampling regions. Figure S8. Ochrophyte genera by sampling regions. Figure S9. HMSC species-to-species co-occurrence and correlation networks for Cercozoan algivores and bacterivores with microalgae, inferred using presence-absence and abundance models. Panels (a–d) depict associations between cercozoan eukaryvores and omnivores with green algae and ochrophytes, while panels (e–h) show associations between cercozoan bacterivores and microalgae. The presence-absen [file 40168_2025_2035_MOESM1_ESM.docx]

Supplementary information

"Enhancing Microbial Predator-Prey Detection with Network and Trait-Based Analyses"

**Authors**:

Cristina Martínez Rendón^1^, Christina Braun^3^, Maria Kappelsberger^4^, Jens Boy^5^, Angélica Casanova-Katny^6^, Karin Glaser^2^, Kenneth Dumack^1*^

^1^Institute of Zoology, Terrestrial Ecology, University of Cologne, ^2^Institute for Biosciences, TU Bergakademie Freiberg, ^3^Institute of Ecology and Evolution, Friedrich Schiller University Jena, ^4^Institute of Planetary Geodesy, Technical University of Dresden, ^5^Institute of Earth System Science, Leibniz Universität Hannover, ^6^Temuco Catholic University.

^*^**Corresponding author**: kenneth.dumack@uni-koeln.de

**Authors' information**

^1^Zülpicher Str. 47b, 50674 Cologne, Germany; c.martinezrendon@uni-koeln.de.

kenneth.dumack@uni-koeln.de.

^2^Leipziger Straße 29, Freiberg, Germany; karin.glaser@ioez.tu-freiberg.de.

^3^Dornburger Str. 159, 07743 Jena, Germany; chr.braun@uni-jena.de.

^4^Helmholtz Str. 10, 01069 Dresden; maria.kappelsberger@tu-dresden.de.

^5^Herrenhäuser Str. 2, 30419 Hannover, Germany; boy@ifbk.uni-hannover.de.

^6^Manuel Montt 56, Temuco, Chile; mcasanova@uct.cl

1. *Sampling sites*

*Svalbard*

Located in the Arctic Ocean, the Svalbard archipelago comprises all islands, islets, and reefs situated between 74° and 81°N, and 10° and 35°E, as defined in the Svalbard Treaty. Spitsbergen, the largest island, is home to the capital Longyearbyen, administrated by Norway [1]. Although entirely within the High Arctic, and with a correspondingly severe climate, Svalbard is considerably milder, wetter, and cloudier than the average for the latitude due to atmospheric heat and moisture transport associated with the Icelandic low and the warm West Spitsbergen Current [2]. The annual mean temperature in Longyearbyen increased from -5.9°C (1971-2000) to -2.5°C (2010-2020), with a mean rainfall of 249 mm in the latter period [3]. This trend is consistent across the entire archipelago, as temperatures have risen by 3 to 5°C in the last decades. Fine-scale simulations project a further temperature increase of 4 to 7°C by 2100 under different scenarios (RCP2.6 and RCP8.5, respectively). Alongside and consequently, the frequency of heavy rainfalls, glacier ice loss, and annual runoff (mainly caused by enhanced glacier melt), have increased [2]. About 16% of Svalbard's land area consists of vegetated peninsulas and valleys [4,5]. The vegetation comprises ca. 204 recorded vascular plants [6], 740 lichens [7], 380 mosses [8], and at least one hundred algal species (Borchhardt, et al., 2017a; Rippin et al., 2018). Permafrost covers the entire landmass of Svalbard, and only the top meter of the earth thaws during the summer. The flora survives in relatively barren ground soil, and is subject to a very short growing season, with periods of continuous daylight and darkness ranging from 120 to 140 days [7].

*King George Island*

The South Shetland archipelago is located north-west of the Antarctic Peninsula, between 61 and 63°S, and 54 and 63°W. Strongly influenced by the Antarctic Circumpolar Current, the region has the mildest climate in Antarctica (Borchhardt, et al., 2017b), characterized by cold, maritime, and moist conditions [12]. The Fildes Peninsula, situated in the southwest of King George, the archipelago's largest island, covers an area of 29 km^2^ [13], and is the island’s largest ice-free zone [14]. The mean annual temperature, recorded in Bellinghausen Station, has increased from -2.3°C (1961-2010) to -1.9°C (2012-2022). Similar to Svalbard, mean annual air temperatures are rising throughout Antarctica, with the most pronounced warming occurring in the Peninsula [15]. In the 2012-2022 period, the mean annual precipitation in the region was 660 mm (NOAA/NCEI; last accessed 09.2023). The vegetation in Fildes consists of 40 described mosses [16], 119 lichens species [17], and two species of flowering plants, namely *Deschampsia antarctica* Desv. and *Colobanthus quitensis* (Kunth) Bart [18]. Moreover, a species richness of 830 algal OTUs was recently described.

*Thala Hills Oasis, Enderby Land*

Enderby Land extends from 38° 30’ E to 57°E and 67°N, and borders the Cosmonauts Sea in the west and the Sea of Cooperation in the east. The Thala Hills (or Molodezhny) encompass the Molodezhnaya base and covers an area of ca. 41 km^2^. They form a coastal oasis with ice-free areas amidst a predominantly glacial landscape, and belong to the low-lying coastal Antarctic oases, common along the coasts of continental Antarctica. The oasis has a more severe climate than others at similar latitudes in East Antarctica. The average air temperature registered at Molodezhnaya station of −11.0 °C (1963-1998, Dolgikh et al., 2015), has persisted in recent years (2017-2022) (NOAA/NCEI; last accessed 09.2023). Soil vegetation cover in the Thala Hills is sparse. Fifty-one lichen and nine moss species are documented [19], while the recorded algae diversity is limited to eight green algae and two diatom species [20].

**Table S4.** Sampling sites

|  | | | Coordinates | |  |
| --- | --- | --- | --- | --- | --- |
| Code | Date | Place | Longitude (°) | Latitude (°) | Observations |
| **Svalbard, Arctic Ocean** | | |  |  |  |
| S1 | 24.07.2021 | Cole mine 200 m | 15.45149326 | 78.236404 | Rocky substrate. Crust dominated by cyanobacteria. Presence of lichens, moss, and small vascular plants. |
| S2 | 24.07.2021 | Seed vault 200 m | 15.480902 | 78.236156 | Meadow. Humid soil. Crust dominated by cyanobacteria. Vascular plants and grass in the periphery. |
| S3 | 25.07.2021 | Small glacier | 15.52066135 | 78.19641113 | Moraine edge next to a mountain with numerous bird nests. Crust dominated by moss; presence of lichens. |
| S4 | 25.07.2021 | Glacier, bird nests | 15.50894356 | 78.18827057 | Moraine. Humid soil. Nests from different bird species. Patches of cyanobacteria, moss, and lichens. |
| S5 | 25.07.2021 | Plateou | 15.5344591 | 78.1935852 | Rocky substrate. Humid soil, very early crust. Patches of moss and plants. |
| S6 | 25.07.2021 | Edge of Longyearbyen | 15.568083 | 78.197576 | Streambank. Very humid soil. Crust dominated by moss. |
| S7 | 28.07.2021 | West valley | 15.33662223 | 78.22031402 | Morain edge. Humid soil. Ptardigans nests. Moss and lichen dominated crust. Presence of vascular plants. |
| S8 | 29.07.2021 | Foxfonna I | 16.06016349 | 78.15654754 | Morain. Humid soil. Crust rich in lichens. Scattered grass and vascular plants. |
| S9 | 29.07.2021 | Foxfonna II | 16.092304 | 78.151367 | Morain. Crust rich in lichens. Scattered grass and vascular plants. |
| **King George Island, Antarctic Peninsula** | | |  |  |  |
| K5 | 29.01.2022 | Kristianka | -58.94074987 | -62.19710322 | Rocky substrate. Wet soil. Crust dominated by cyanobacteria. Presence of moss. |
| K6 | 29.02.2022 | Suffield Point | -58.92696496 | -62.19073175 | Morain. Developed crusts, patches of moss and lichens. |
| K7 | 06.02.2022 | Meseta Cruz | -58.95625653 | -62.20711263 | Rocky substrate. Developed crusts, patches of moss and lichens. |
| K9 | 12.02.2022 | Strand Bellingshausen | -58.95652128 | -62.19812962 | Clayey soil. Crust dominated by moss in patches. |
| C2 | 26.02.2022 | Collins 2 | -58.85636111 | -62.16944444 | Developed crusts with moss and lichens. |
| C3 | 26.02.2022 | Collins 3 | -58.85288889 | -62.16977778 | Developed crusts with moss and lichens. |
| C4 | 26.02.2022 | Collins 4 | -58.85147222 | -62.16966667 | Developed crusts with moss and lichens. |
| Me1 | 07.03.2022 | Meseta 1 | -58.92666333 | -62.176915 | Sandy and rocky substrate. Crust dominated by lichens. Presence of moss. |
| Me2 | 07.03.2022 | Meseta 2 | -58.92400833 | -62.174105 | Presence of lichens and scattered moss. |
| Me3 | 07.03.2022 | Meseta 3 | -58.93071333 | -62.17876833 | Crust dominated by moss. |
| **Thala Hills, Continental Antarctica** | | |  |  |  |
| BIO1 | 07.02.2022 | Molodezhnaya | 45.86073 | -67.66765 | Rocky and sandy soil. Young biocrust with presence of lichens. |
| BIO2 | 07.02.2022 | Ovalnoye-Glubokoye | 45.8684 | -67.66871 | Rocky and sandy soil. Crust dominated by lichens. |
| BIO3 | 08.02.2022 | Sandiger See | 45.85368333 | -67.65753333 | Sandy soil. Scattered areas with thowing snow. Moss dominated crust. Colorful lichens. |
| BIO4 | 10.02.2022 | Blisnetzow | 45.91948333 | -67.66286667 | Rocky and sandy soil. Scattered moss. |

1. *Primer design*

The use of universal eukaryotic primers in environmental sequencing typically leads to libraries that inadequately represent the true microbial diversity. Certain genetic loci may not efficiently capture all taxonomic members or exhibit biases [21], often producing non-saturated data. Therefore, we developed taxon-specific primers for green algae and diatoms. A metabarcoding approach using tagged versions of the primers (Tables S5 and S6 for tag details), was chosen as it enables the simultaneous analysis of multiple samples while reducing costs. Furthermore, it allows a targeted analysis and ensures data saturation, taxonomic resolution, and sensitivity. The method can potentially detect low-abundance taxa, which may not be uncovered by other omics techniques that offer broader but less sensitive insights into microbiomes. Our protocol development followed the methodologies applied by Fiore-Donno et al. (2018) in designing Cercozoa-specific primers.

We targeted the V4 region of the 18S rRNA-encoding gene due to its suitability as a marker region for diatom analyses [22,23]. Although no single marker has yet proven ideal across all green algae lineages [21], while having a length suited for Illumina MiSeq sequencing, and a rich sequence-library availability, we applied 18S rRNA gene sequencing as it provides sufficient coverage and insight into terrestrial taxa at genus level. Universal eukaryotic primers [24], primers for green algae [25–27], and diatoms [22,23,28] were tested for specificity and coverage using TestPrime 1.0 on the non-redundant SILVA database r138.1 [29]. We selected one literature primer (Euk1, Van Borm & Boomsma, 2002), along with modified and novel primers (Table S7). The protocol developed for diatoms proved highly efficient, retrieving *in silico* 66% of the terrestrial diatoms listed in SILVA. It demonstrated improved recovery rates of certain terrestrial taxa, such as Bacillariophycea (4.7%) and Mediophycea (9.6%), when compared to the approach developed by Visco et al. (2015). The protocol for green algae demonstrated to be very specific, effectively targeting most terrestrial green algae, with the exception of one taxon, Klebsormidiophycea (Charophyta), which allocates a few terrestrial species. *In silico,* 48% of the chlorophytes within SILVA were targeted with zero mismatches, while only targeting less than 5% of the embryophytes in the database (see Figures S1 and S2, *In silico* results).

**Top of Form**

**Table S5.** Diatom primers and tags designed for this study*

|  |  | Forward 5'-3' |  | Reverse 5'-3' |
| --- | --- | --- | --- | --- |
| Primer | *EukF1* | AGCAGCCGCGGTAATTCC | *Diat_Rv1* | CCTCTGACAATGRAATACGAATAC |
| Tag | A | TTCTATCG | A | TACTTAGC |
|  | B | TAGTGAGA | B | TCTTAGTG |
|  | C | TCCTCAAT | C | TCTCTTAG |
|  | D | TCGTACCT | D | AACAATGC |
|  | E | TGATTGAG | E | AACCTTAG |
|  | F | TGGACTAT | F | AAGTTACG |
|  | G | ACACACCT | G | CTGAATAG |
|  | H | AGCAGATA | H | CGAATGCC |
|  | I | CTAACGTA | I | GTGAACCG |
|  | J | CATACTCA | J | GTGCTACG |
|  | K | GAAGCGTT | K | GCTTACCG |
|  | L | GACCGGAA | L | GCGAAGTG |

*Twelve barcoded versions for *EukF1* and 12 for *Diat_Rv1* allow 144 possible combinations.

**Table S6.** Green algae primers and tags designed for this study*

|  |  | Forward 5'-3' |  | Reverse 5'-3' |
| --- | --- | --- | --- | --- |
| Primer | *EukF1* | AGCAGCCGCGGTAATTCC | *GreenAl_RV1* | CTGACAAYGAAATACGAATGC |
| Tag | A | TTATGAGG | A | TTACATCC |
|  | B | TATCCACT | B | TAATACGC |
|  | C | TAGGATTG | C | TCGTCACG |
|  | D | TCGCTGTT | D | TGTTCGCA |
|  | E | TGATGGTA | E | TGCCGTCA |
|  | F | ATTCACCT | F | TGGTCTTA |
|  | G | ATAGGACT | G | AACCAAGA |
|  | H | AATCTGCA | H | AAGGACCA |
|  | I | AAGTTGCG | I | AGACACGG |
|  | J | AAGAAGTG | J | CTTGTATG |
|  | K | ACATACCT | K | CTTGACTA |
|  | L | ACGCCTAA | L | CAAGGCAC |
|  | M | CAATTCGA | M | GATGCAGC |
|  | N | CCTATCTA | N | GCACTCAA |
|  | O | GAGTCGTT | O | GCACAACC |

*Fifteen barcoded versions for GreenAl_RV1 and 15 for EukF1 allow 225 possible combinations.

**Table S7.** Primers used in this study

| Taxon | Semi-nested PCR | Primers | | Ta^+^ °C | ≈ Length (bp) |
| --- | --- | --- | --- | --- | --- |
|  |  | Forward (5’-3’) | Reverse (5’-3’) |  |  |
| **Cercozoa^1^** | 1^ST^ PCR | S616F_Cerco^1^: TTAAAAAGCTCGTAGTTG | S963R_Cerco^1^: CAACTTTCGTTCTTGATTAAA | 50 |  |
|  | 2^ND^ PCR | S616F_Cerco^1^ | S947R_Cerco^1^: AAGAAGACATCCTTGGTG | 50 | 320-345 |
| **Diatoms** | 1^ST^ PCR | EukF1^2^:  AGCAGCCGCGGTAATTCC | Diat_Rv2^3^:  CACCTCTGACAATGRAATAC | 64 |  |
|  | 2^ND^ PCR | EukF1^2^ | Diat_Rv1^3^:  CCTCTGACAATGRAATACGAATAC | 64 | 290-358 |
| **Green algae** | 1^ST^ PCR | EukF1^2^ | Chlphy_RV1^3^:  GACTAKGACGGTATCTAA | 58 |  |
|  | 2^ND^ PCR | EukF1^2^ | GreenAl_RV1^3^:  CTGACAAYGAAATACGAATGC | 56 | 304-383 |
| ^+^Ta: annealing temperature; ^1^Fiore-Donno et al., 2018; ^2^Van Borm & Boomsma, 2002; ^3^This study. | | | | | |


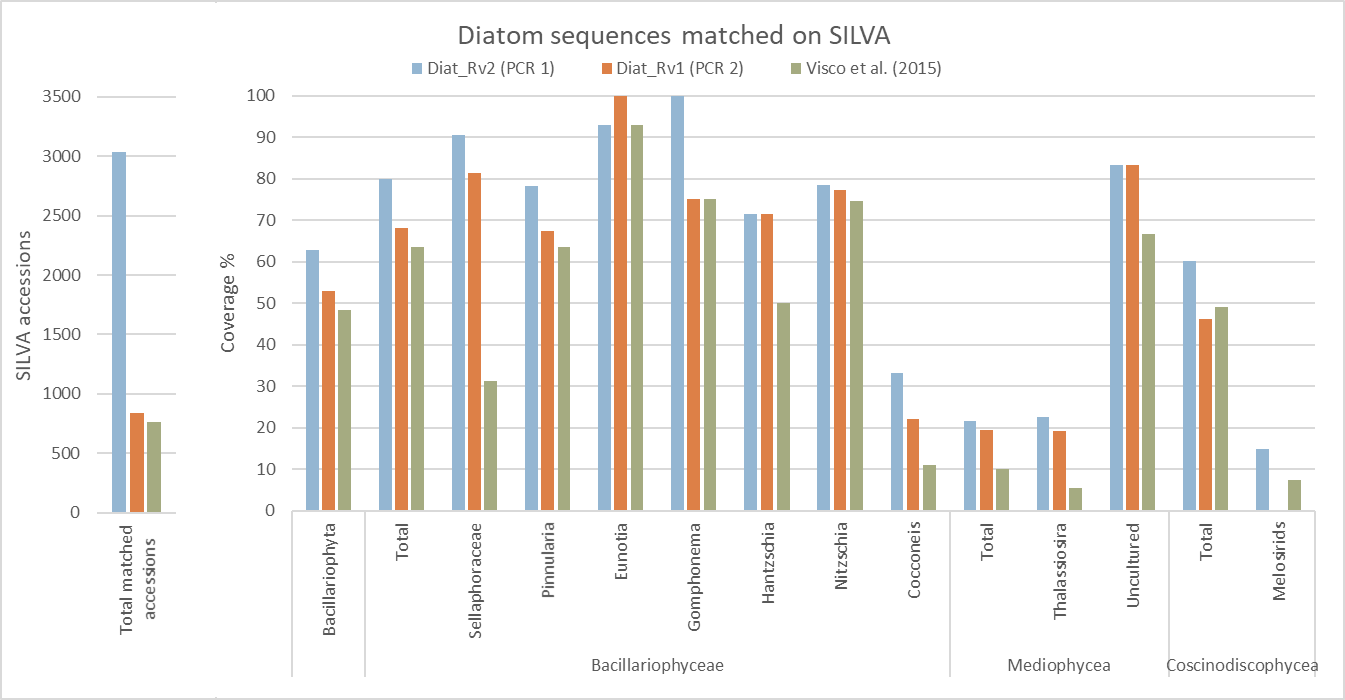


a.

b.

**Figure S1.** ***In silico* comparison of amplification efficiency of diatom designed and literature primers**.

All reactions were conducted with the forward primer EukF1. a. Total matched accessions of every tested PCR. The non-redundant SILVA database had 1,571 sequences within the Bacillariophyta at the time of the study. b. Major terrestrial taxa of the three diatom classes are presented. Results are based on a perfect match (0 mismatches) on the SILVA database.


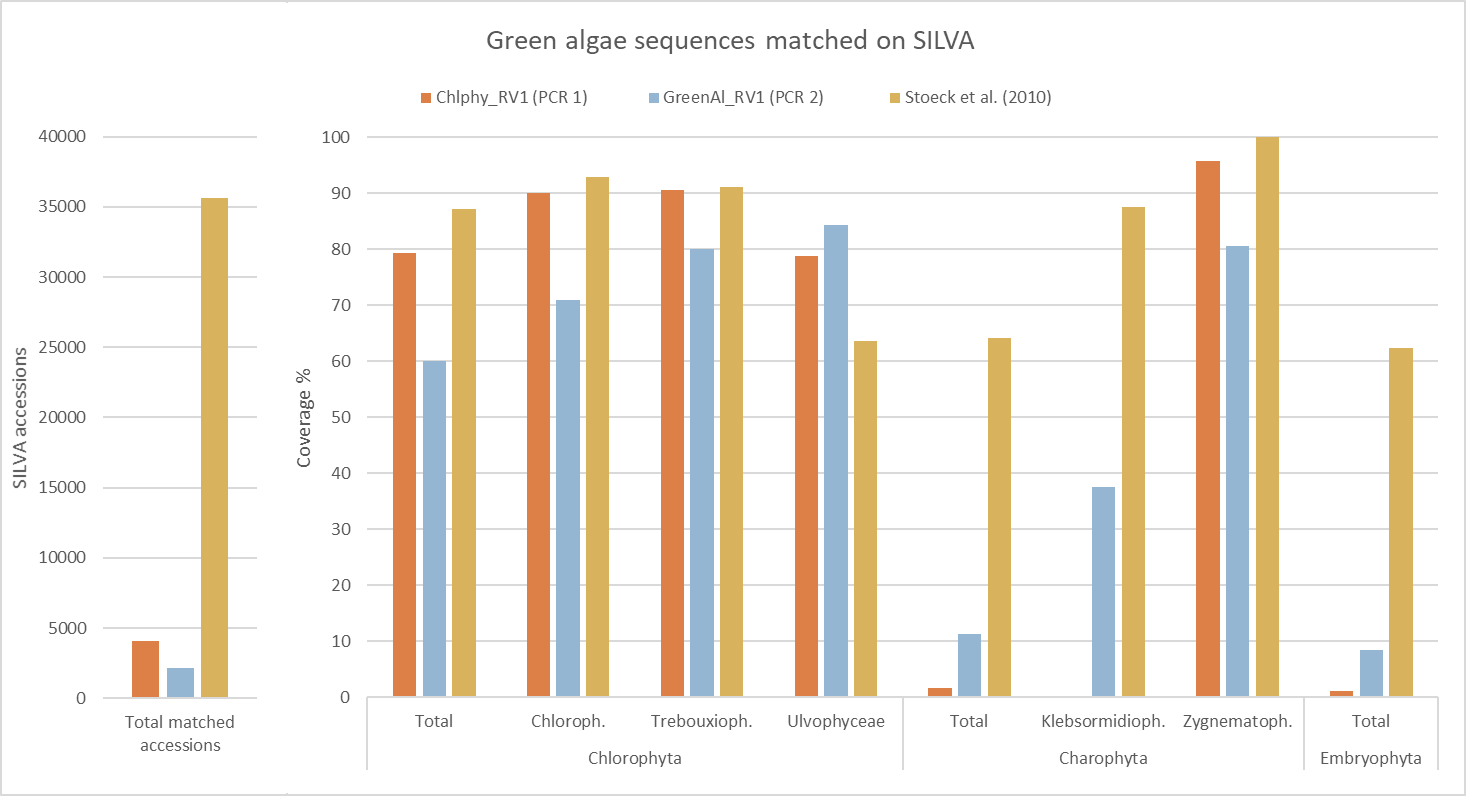


a.

b.

**Figure S2. *In silico* comparison of amplification efficiency of green algae primers**. All reactions were conducted with the forward primer EukF1. a. Total matched accessions of every tested PCR. The non-redundant SILVA database had 1,908 Chlorophyta and 4,738 Charophyta sequences at the time of the study. The compared methodology [27] comprise general eukaryotic primers, which expectedly matched a large number of eukaryotic accessions. b. Major terrestrial microalgae taxa are shown within each phylum. Total Embryophyta matched accessions are also shown. Since the developed protocol was intended for microalgae analysis, we aimed to develop specific primers to avoid the amplification embryophytes. Results are based on a perfect match (0 mismatches) on the SILVA database.

1. *Internal standard: mock communities*

As an internal standard, mock communities were sequenced together with amplicon pools. DNA was extracted from dense cultures of green algae and ochrophytes originating from this study and was pooled in equal concentrations to prepare mock communities for each taxon. The selected taxa were carefully screened to ensure a minimum dissimilarity of 97% in the sequenced fragment, a threshold often regarded as providing robust species-level resolution during OTU clustering. DNA from Cercozoa taxa was obtained from a private culture collection of the Terrestrial Ecology, University of Cologne (Table S8). All taxa were subjected to the second PCR scheme with tagged primers, purified, quantified, and normalized before adding each mock community as a sample to its respective library.

**Table S8.** Cercozoa, green algae, and ochrophytes included in the internal standards of each run

| **ID** | **Taxonomic assignment** | **Genus/Species** | **Provided by** |
| --- | --- | --- | --- |
| **Cercozoa** |  |  |  |
| K92 | Imbricatea/Krakenida | *Kraken carinae* | Terrestrial Ecology, Cologne University |
| CCAP1943 | Thecofilosea/Tectofilosida | *Fisculla terrestris* | Terrestrial Ecology, Cologne University |
| RC | Thecofilosea/Cryomonadida | *Rhogostoma pseudocylindrica* | Terrestrial Ecology, Cologne University |
| M01 | Thecofilosea/Cryomonadida | *Rhogostoma minus* | Terrestrial Ecology, Cologne University |
| C12 | Sarcomonadea/Glissomonadida | Sandonid | Terrestrial Ecology, Cologne University |
| C70 | Sarcomonadea/Glissomonadida | Sandonid | Terrestrial Ecology, Cologne University |
| C4 | Sarcomonadea/Glissomonadida | Allapsid | Terrestrial Ecology, Cologne University |
| C31 | Sarc./Paracercomonadida | Paracercomonad | Terrestrial Ecology, Cologne University |
| C15 | Sarc./Cercomonadida | Neocercomonad | Terrestrial Ecology, Cologne University |
| **Green algae** | |  |  |
| 1EA6 | Chlorophycea/Sphaeropleales | *Bracteacoccus bullatus* | This study |
| 1EA9 | Chlorophycea/Sphaeropleales | *Neocystis brevis* | This study |
| A10 | Chlorophycea/Sphaeropleales | *Coelastrella oocystiformis* | This study |
| 9BA1 | Chlorophycea/Chlamydomonadales | *Chlamydomonas/Chloromonas* | This study |
| 1EA12 | Trebouxiophyceae/Prasiolales | *Deuterostichococcus epilithicus* | This study |
| A2.1 | Trebouxiophyceae/Chlorellales | *Auxenochlorella sp.* | This study |
| A9.1 | Trebouxiophyceae/Trebouxiales | *Myrmecia bisecta* | This study |
| 7CA3 | Klebsormidiophyceae/  Klebsormidiales | *Klebsormidium elegans* | This study |
| **Ochrophytes** | |  |  |
| 7CA4 | Bacillariophyceae/Naviculales | *Pinnularia borealis* | This study |
| 3EA8 | Bacillariophyceae/Naviculales | *Pinnularia* sp. | This study |
| 7CA12 | Bacillariophyceae/Naviculales | *Sellaphora seminulum* | This study |
| 7CA9 | Bacillariophyceae/Bacillariales | *Nitzschia perminuta* | This study |

1. *Cultures*

**Table S9. Cercozoa cultures established in this study.**

|  | **Code** | **Genus** | **species** | **Class** | **Matched accession** | **Percent identity** | **Geographic origin** | **Region** |
| --- | --- | --- | --- | --- | --- | --- | --- | --- |
| 1 | 1AH1* | *Euglypha* | *rotunda* | Imbricatea | KP728379.1 | 97.22 | Svalbard | Arctic |
| 2 | 3EH1 | *Neocercomonas/*  *Cercomonas* | sp.  *plasmodialis* |  | MG775618.1/  AF411268.1 | 95.15 | Svalbard | Arctic |
| 3 | 3EH3 | *Rhogostoma* | *schussleri* | Thecofilosea | HQ121430.1 | 99.34 | Svalbard | Arctic |
| 4 | 4BH1 | *Neocercomonas/*  *Cercomonas* | sp./  sp. | Cercomonadidae | MG775599.1/ HM536151.1 | 100.00 | Svalbard | Arctic |
| 5 | 7CH7 | *Fisculla* | *terrestris* | Thecofilosea | KP728379.1 | 98.77 | Svalbard | Arctic |
| 6 | 9BH5* | *Fisculla* | *nemoris* | Thecofilosea | KT809361.1 | 100 | Svalbard | Arctic |
| 7 | C2.2 H5* | *Cercomonas* | *celer* | Cercomonadidae | FJ790710 | 99.04 | King George Island | Antarctic Peninsula |
| 8 | C2.2 H6* | *Cercomonas* | *plasmodialis* | Cercomonadidae | AF411268 | 99.68 | King George Island | Antarctic Peninsula |
| 9 | C2.2 H7 | *Neocercomonas* | *tuberculata* | Cercomonadidae | MG775594 | 98.12 | King George Island | Antarctic Peninsula |
| 10 | B2.5 H2 | *Sandona* | *limna* | Sandonidae | HQ918177 | 100 | Molodezhnaya | Continental Antarctica |
| 11 | B2.5 H3 | *Bodomorpha* | sp. | Viridiraptoridae | DQ211596 | 99.37 | Molodezhnaya | Continental Antarctica |
| 12 | B2.5 H4 | *Spongomonas* | *minima* | Imbricatea | AF411280 | 98.03 | Molodezhnaya | Continental Antarctica |
| 13 | B2.5 H6 | *Cercomonas* | *plasmodialis* | Cercomonadidae | AF411268 | 100 | Molodezhnaya | Continental Antarctica |
| 14 | B2.5 H9 | *Euglypha* | *rotunda* | Imbricatea | AJ418784 | 99.58 | Molodezhnaya | Continental Antarctica |
| 15 | B3.3 H1 | *Rhogostoma* | *epiphylla* | Thecofilosea | KY905095 | 99.69 | Molodezhnaya | Continental Antarctica |
| 16 | B3.5 H2* | *Rhogostoma* | *schussleri* | Thecofilosea | HQ121430.1 | 99.00 | Molodezhnaya | Continental Antarctica |
| 17 | B3.5 H1 | *Neocercomonas* | sp. | Cercomonadidae | MG775612.1 | 99.68 | Molodezhnaya | Continental Antarctica |
| 18 | B4.2 H2 | *Rhogostoma* | *epiphylla* | Thecofilosea | KY905095.1 | 98.37 | Molodezhnaya | Continental Antarctica |
| 19 | B4.2 H4 | *Cercomonas* | *plasmodialis* | Cercomonadidae | AF411268.1 | 99.67 | Molodezhnaya | Continental Antarctica |
| 20 | B4.2 H5 | *Euglypha* | *rotunda* | Imbricatea | AJ418784.1 | 100 | Molodezhnaya | Continental Antarctica |
| 21 | B4.2 H7 | *Heteromita* | sp. | Sarcomonadea | HM536169.1 | 100 | Molodezhnaya | Continental Antarctica |

*Cultures used in the validation of network associations.

**Table S10. Algae and ochrophyte cultures established in this study.**

|  | **Code** | **Genus** | **species** | **Class** | **Matched accession** | **Percent identity** | **Geographic origin** | **Region** |
| --- | --- | --- | --- | --- | --- | --- | --- | --- |
| 1 | 3EA8 | *Pinnularia* | sp. | Bacillariophyceae | KM350088.1 | 99.31 | Svalbard | Arctic |
| 2 | 7CA11 | *Sellaphora* | *seminulum* | Bacillariophyceae | CP024866.1 | 78.99 | Svalbard | Arctic |
| 3 | 7CA4 | *Pinnularia* | *borealis* | Bacillariophyceae | MN940518.1 | 99.28 | Svalbard | Arctic |
| 4 | 7CA9 | *Nitzschia* | *perminuta* | Bacillariophyceae | MN696726.1 | 99.64 | Svalbard | Arctic |
| 5 | 1EA1 | *Gloeocystis* | sp. | Chlorophyceae | GU117588.1 | 98.17 | Svalbard | Arctic |
| 6 | 1EA10 | Radiococcaceae | sp. | Chlorophyceae | JX169836.1 | 96.72 | Svalbard | Arctic |
| 7 | 1EA11 | *Chlamydomonas* | *moewusii* | Chlorophyceae | FR865525.1 | 98.93 | Svalbard | Arctic |
| 8 | 1EA6 | *Bracteacoccus* | *bullatus* | Chlorophyceae | MG582205.1 | 98.83 | Svalbard | Arctic |
| 9 | 3EA3 | *Dictyococcus* | *varians* | Chlorophyceae | KF673359.21/  HQ292768.1 | 98.55 | Svalbard | Arctic |
| 10 | 3EA5 | *Bracteacoccus* | *bullatus* | Chlorophyceae | MG582205.1 | 95.29 | Svalbard | Arctic |
| 11 | 3EA6 | *Dictyococcus/*  *Pseudomuriella* | sp. | Chlorophyceae | KF673359.1/  MW075327.1 | 98.04 | Svalbard | Arctic |
| 12 | 6DA1 | Haematococcaceae |  | Chlorophyceae | EF023280.1 | 98.56 | Svalbard | Arctic |
| 13 | 7CA21 | *Chlamydomonas/*  *Chloromonas* | sp. | Chlorophyceae | LC639358.1/  MT735204.1 | 98.34 | Svalbard | Arctic |
| 14 | 9BA1 | *Chlamydomonas/*  *Chloromonas* | sp. | Chlorophyceae | MG022664.1/  MK912145.1 | 100.0/  100.0 | Svalbard | Arctic |
| 15 | 7CA15 | *Vischeria* | *vischeri/*  *punctata* | Eustigmatophyceae | KM020046.1/  EU878374.1 | 99.26/  99.26 | Svalbard | Arctic |
| 16 | 1EA2 | *Klebsormidium* | *elegans* | Klebsormidiophyceae | MK262904.1 | 98.95 | Svalbard | Arctic |
| 17 | 7CA3 | *Klebsormidium* | *elegans* | Klebsormidiophyceae | MK262904.1 | 95.02 | Svalbard | Arctic |
| 18 | 7CA8 | *Tetrasporopsis* | *moei* | Phaeosacciophyceae | MT582122.1 | 98.34 | Svalbard | Arctic |
| 19 | 1EA12 | *Deuterostichococcus* | *epilithicus* | Trebouxiophyceae | MT078166.1 | 99.08 | Svalbard | Arctic |
| 20 | 1EA13 | Chlorellales | sp. | Trebouxiophyceae | KX094778.1 | 98.59 | Svalbard | Arctic |
| 21 | 1EA14 | *Coccomyxa* | *subellipsoidea* | Trebouxiophyceae | HG972973.1 | 100.00 | Svalbard | Arctic |
| 22 | 1EA16 | Chlorellales | sp. | Trebouxiophyceae | KX094778.1 | 100.00 | Svalbard | Arctic |
| 23 | 1EA8 | Chlorellales | sp. | Trebouxiophyceae | KX094778.1 | 98.58 | Svalbard | Arctic |
| 24 | 7CA12 | *Lobosphaera/*  *Parietochloris* | sp. | Trebouxiophyceae | KT072976.1 | 94.47 | Svalbard | Arctic |
| 25 | 7CA16 | *Parietochloris* | *bilobata/*  *pseudoalveolaris* | Trebouxiophyceae | FJ858973.1/  FJ858971.1 | 98.97/  98.97 | Svalbard | Arctic |
| 26 | 7CA6 | *Coccomyxa* | *subellipsoidea* | Trebouxiophyceae | AC277064.1 | 98.68 | Svalbard | Arctic |
| 27 | 7CA7 | *Chlorella* | *lewinii* | Trebouxiophyceae | FM205861.1 | 95.31 | Svalbard | Arctic |
| 28 | 1EA15 | *Pleurochloris* | *meiringensis* | Xanthophyceae | AJ579340.1 | 97.55 | Svalbard | Arctic |
| 29 | 1EA17 | *Heterococcus/*  *Mischococcus* | sp. | Xanthophyceae | AM490820.1/  AF083400.1 | 93.87/  93.87 | Svalbard | Arctic |
| 30 | 1EA18 | *Excentrochloris/*  *Botrydiopsis* | sp. | Xanthophyceae | HE573277.1/  AJ579339.1 | 98.96/  98.96 | Svalbard | Arctic |
| 31 | 1EA7 | *Botrydiopsis* | *constricta* | Xanthophyceae | AJ579339.1 | 98.96 | Svalbard | Arctic |
| 32 | 3EA2 | *Heterococcus* | *caespitosus/*  *protonematoides* | Xanthophyceae | AM490820.1/  AJ579334.1 | 97.39 | Svalbard | Arctic |
| 33 | 3EA7 | *Xanthonema* | sp. | Xanthophyceae | AM491615.1 | 97.49 | Svalbard | Arctic |
| 34 | 7CA1 | *Xanthonema* | sp. | Xanthophyceae | AM491615.1 | 98.24 | Svalbard | Arctic |
| 35 | 7CA2 | *Tribonema* | *minus* | Xanthophyceae | MT809603.1 | 98.22 | Svalbard | Arctic |
| 36 | 7CA5 | *Cylindrocystis* | *brebissonii* | Zygnemophyceae | MT901370.1 | 99.66 | Svalbard | Arctic |
| 37 | C 2.2 A10 | *Coelastrella* | *oocystiformis* | Chlorophyceae | MH176105 | 100 | King George Island | Antarctic Peninsula |
| 38 | C 2.2 A11 | *Macrochloris* | *radiosa* | Chlorophyceae | KM020104 | 100 | King George Island | Antarctic Peninsula |
| 39 | C 2.2 A8 | *Chlamydomonas* | *mutabilis* | Chlorophyceae | AB701537 | 97.25 | King George Island | Antarctic Peninsula |
| 40 | C 2.2 A12 | *Leptosira* | *erumpens* | Trebouxiophyceae | Z68696 | 99.66 | King George Island | Antarctic Peninsula |
| 41 | C 2.2 A2 | *Auxenochlorella* | sp. | Trebouxiophyceae | KP081390 | 99.36 | King George Island | Antarctic Peninsula |
| 42 | C 2.2 A4 | *Coccomyxa* | *subellipsoidea* | Trebouxiophyceae | HG972973 | 100 | King George Island | Antarctic Peninsula |
| 43 | C 2.2 A6 | *Stichococcus* | sp. | Trebouxiophyceae | KX094817 | 98.98 | King George Island | Antarctic Peninsula |
| 44 | C 2.2 A6 | *Stichococcus* | sp. | Trebouxiophyceae | KX094817 | 98.98 | King George Island | Antarctic Peninsula |
| 45 | C 2.2 A9 | *Myrmecia* | *bisecta* | Trebouxiophyceae | LC366918 | 100 | King George Island | Antarctic Peninsula |
| 46 | K 5.2 A5 | *Chlorella* | sp. | Trebouxiophyceae | MN960178.1 | 98.34 | King George Island | Antarctic Peninsula |
| 47 | B 1.3 A6 | Chlamydomonadales | sp. | Chlorophyceae |  |  | Molodezhnaya | Continental Antarctica |
| 48 | B 2.5 A3 | *Macrochloris* | *rubrioleum* | Chlorophyceae | AB983624 | 100 | Molodezhnaya | Continental Antarctica |
| 49 | B 2.5 A7 | *Bracteacoccus* | *bullatus* | Chlorophyceae | MG582205 | 100 | Molodezhnaya | Continental Antarctica |
| 50 | B 4.2 A1 | *Bracteacoccus* | *bullatus* | Chlorophyceae | AF513377.1 | 100 | Molodezhnaya | Continental Antarctica |
| 51 | B 3.5 A5 | *Chlorella* | sp. | Chlorophyceae | MN960178.1 | 98.34 | Molodezhnaya | Continental Antarctica |
| 52 | B 2.5 A1 | Chlorellales | sp. | Trebouxiophyceae | KX094778 | 96.94 | Molodezhnaya | Continental Antarctica |

1. *Rarefaction curves*


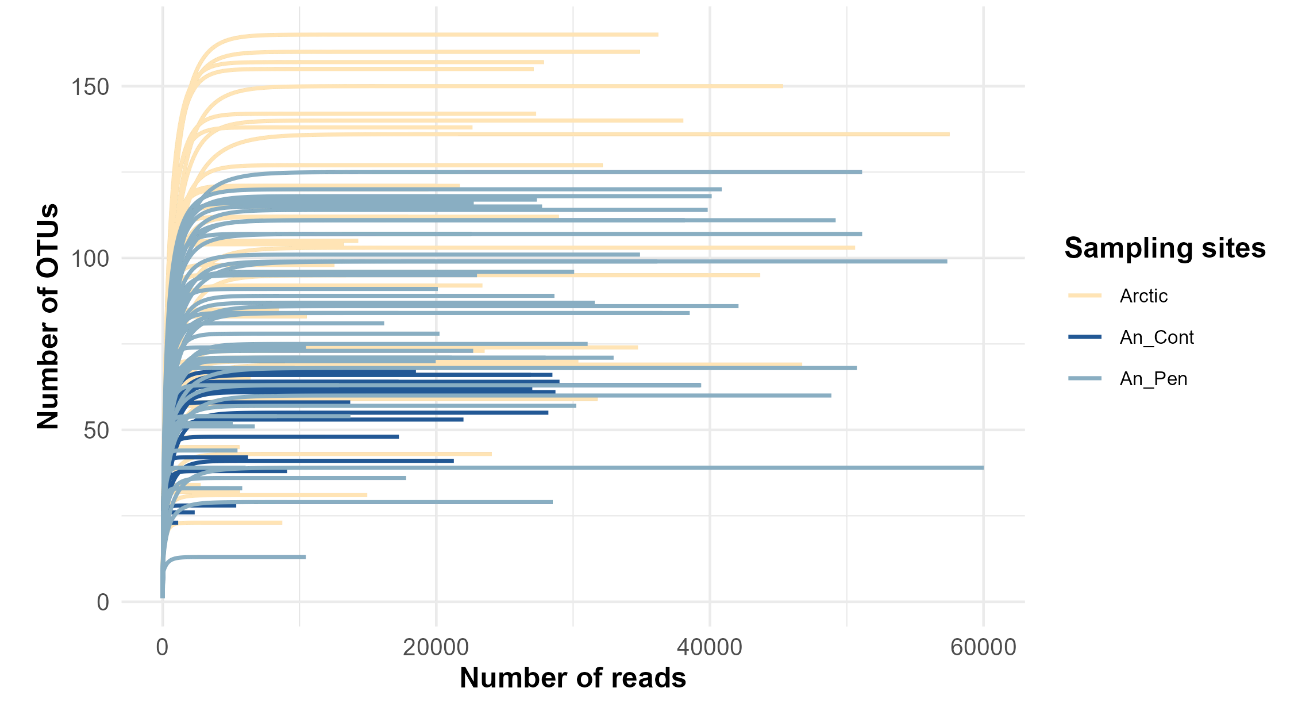


**Figure S3.** Rarefaction curves per sampling sites, Cercozoa


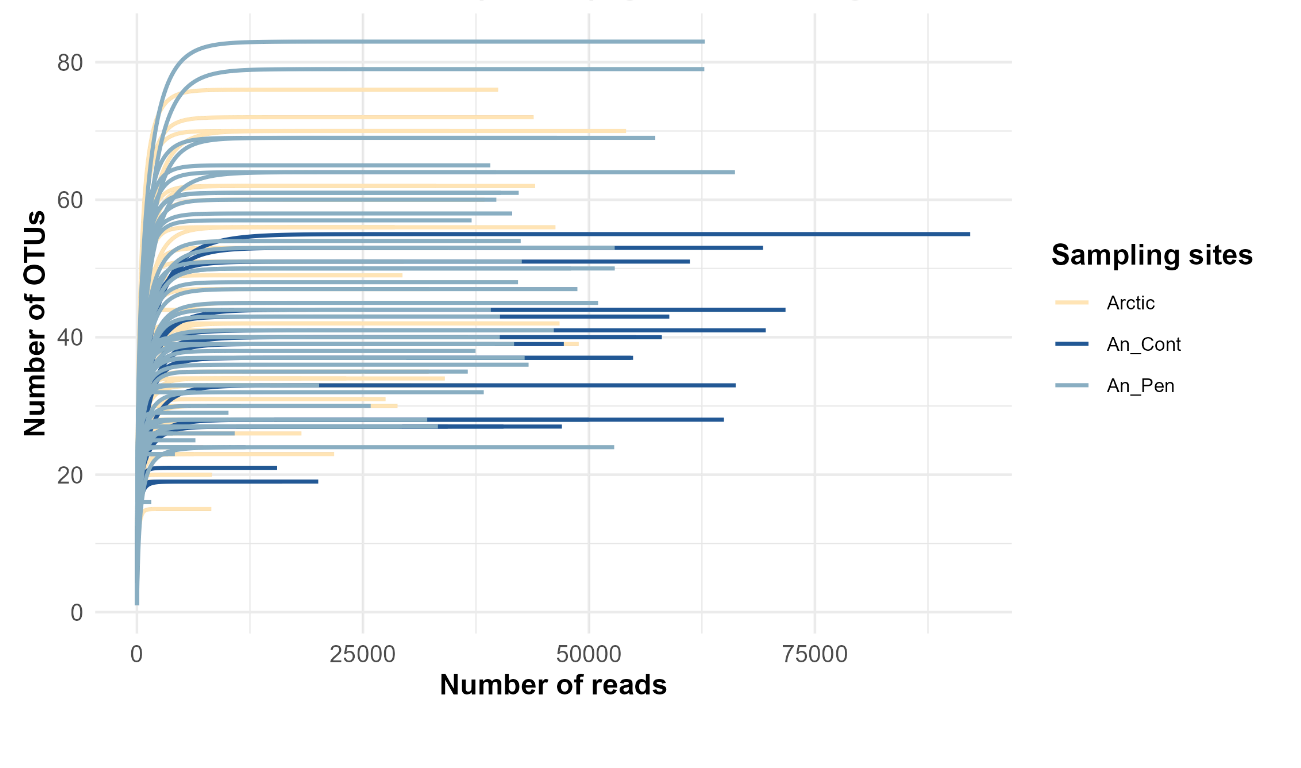


**Figure S4.** Rarefaction curves per sampling sites, green algae


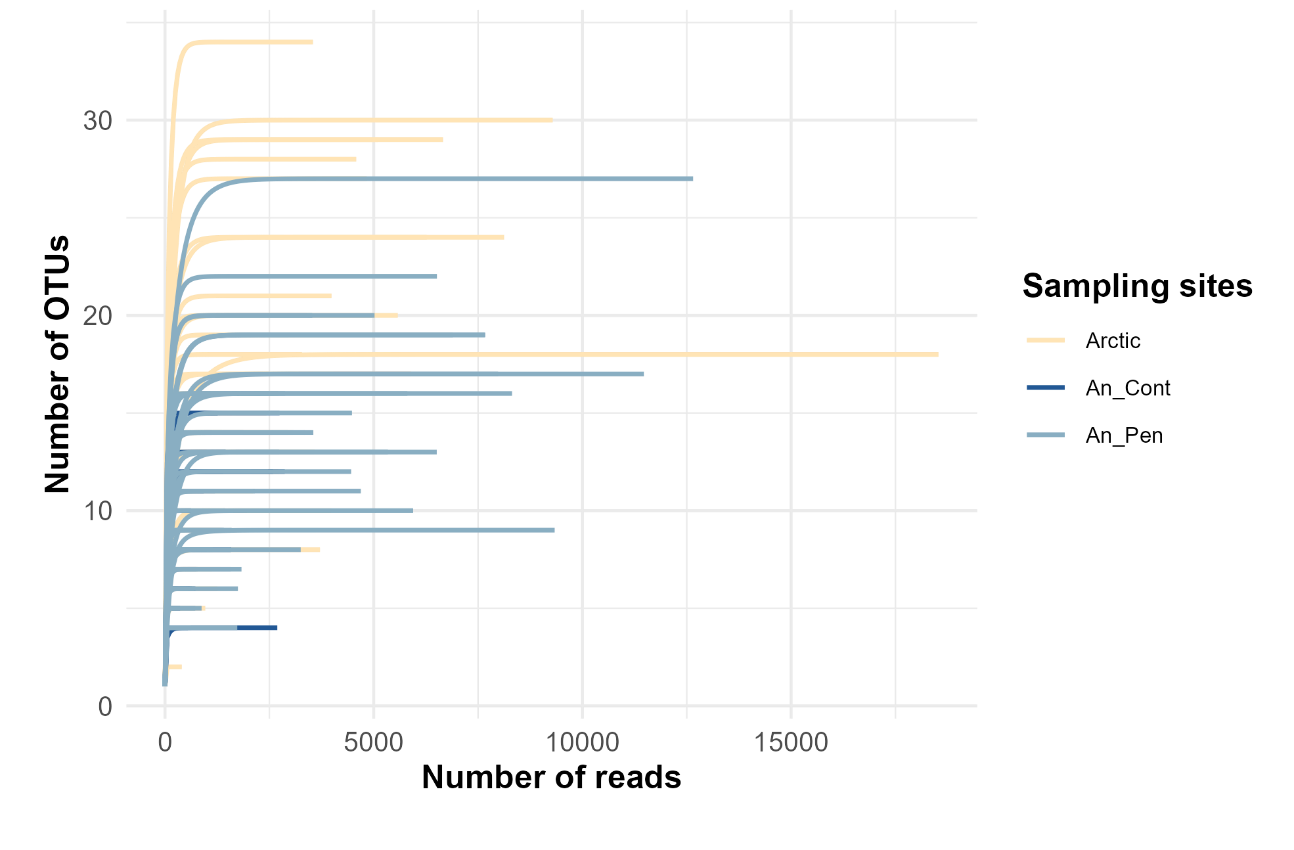

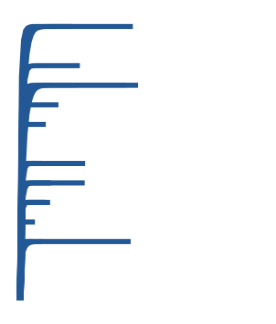


**Figure S5.** Rarefaction curves per sampling sites, ochrophytes

*6. Chord diagrams with genera names*


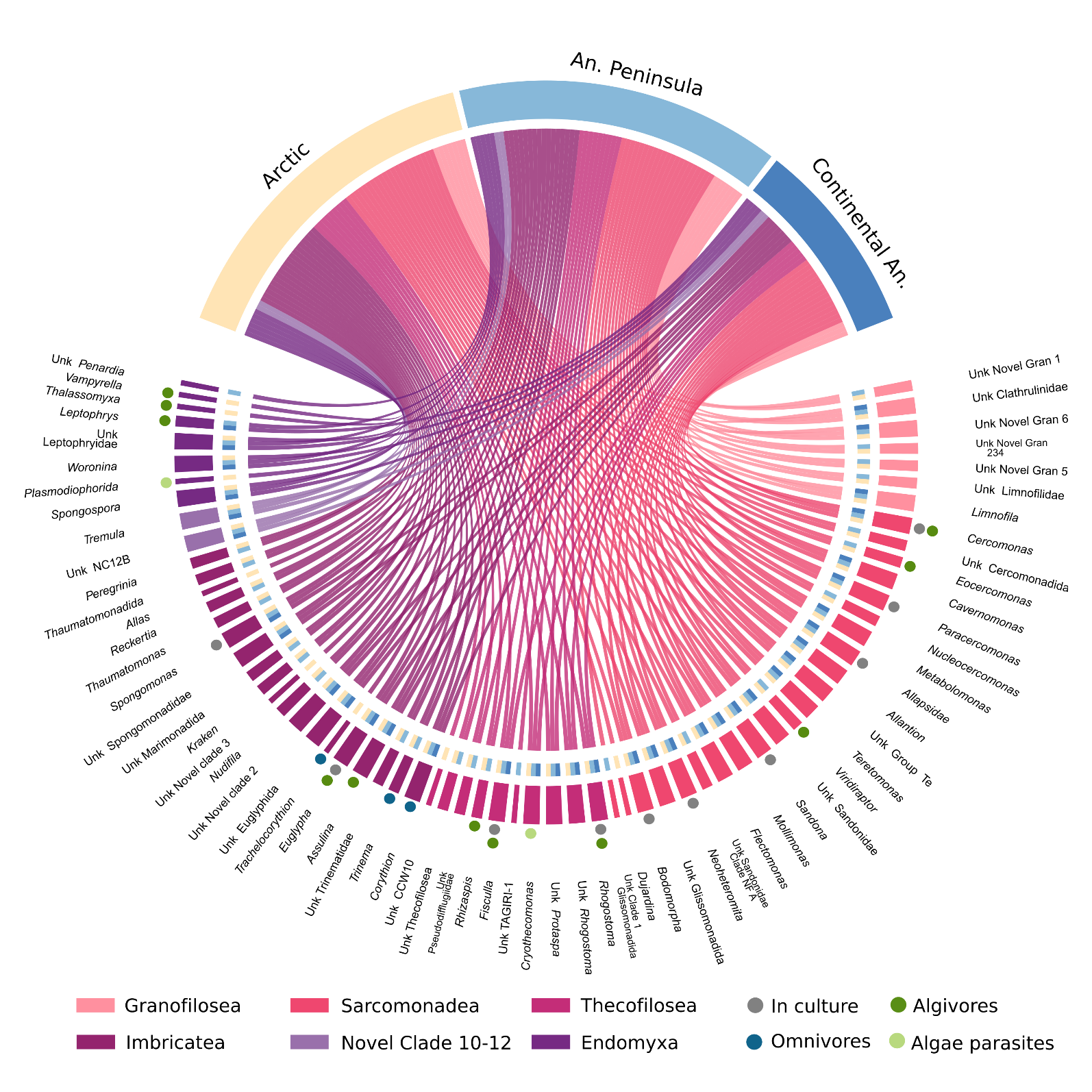


**Figure S6.** Cercozoan genera by sampling regions


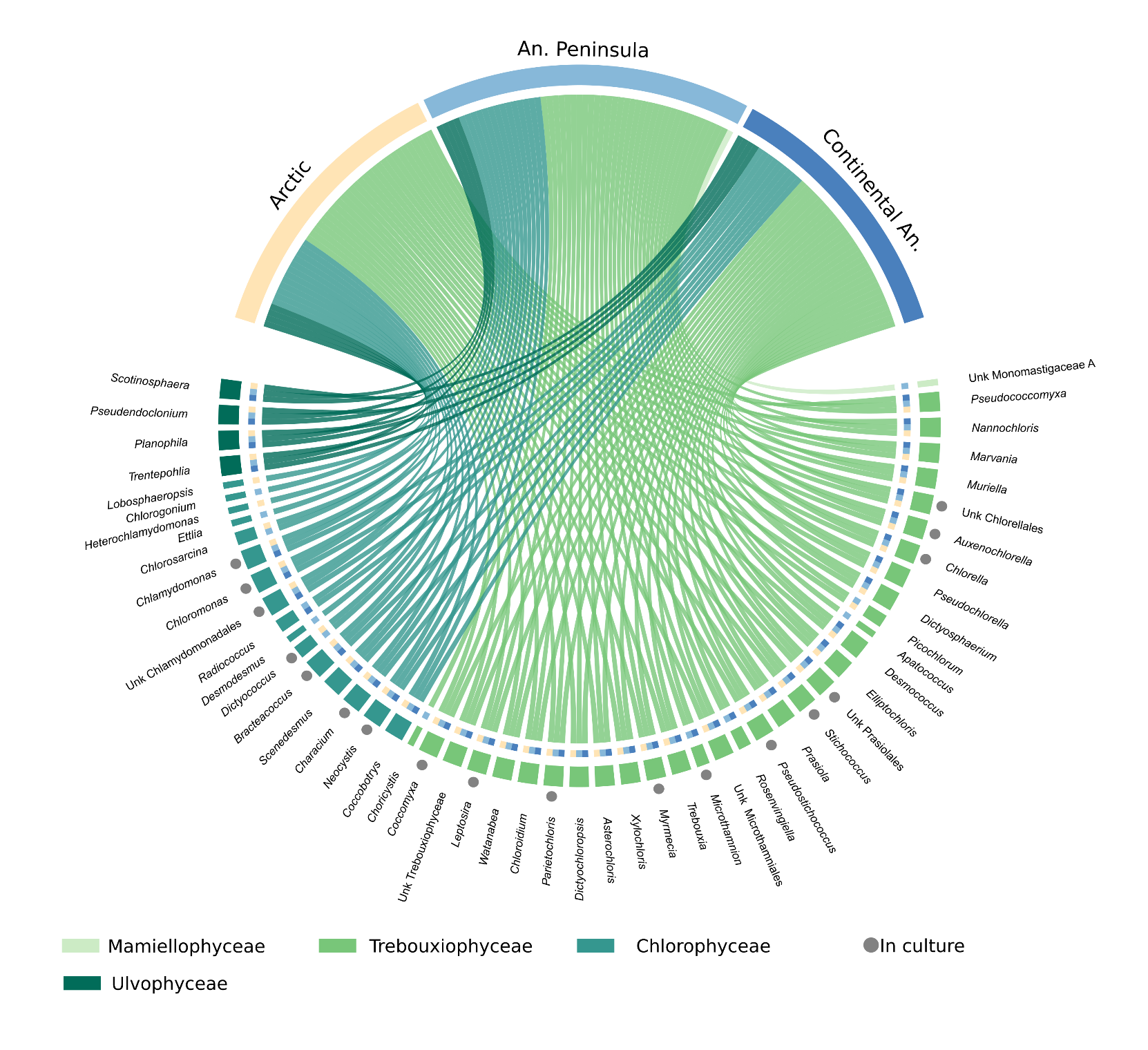


**Figure S7.** Green algal genera by sampling regions


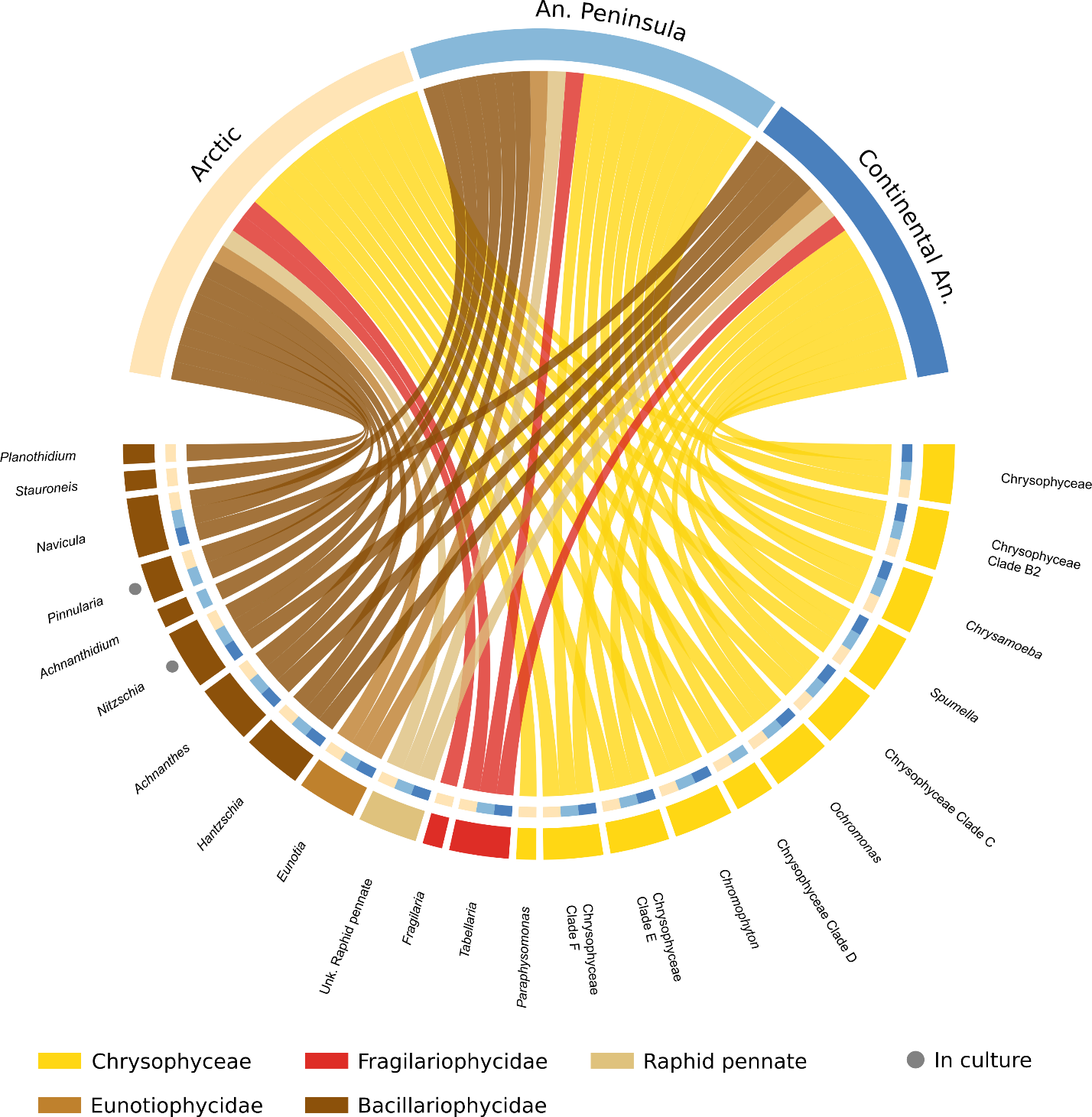


**Figure S8.** Ochrophyte genera by sampling regions

*7. Alpha and Beta diversity statistics*

**Table S11. One-way ANOVA comparisons of alpha diversity indices** — OTU richness, exponential Shannon, and inverse Simpson— across polar biocrusts samples (N=116) from Svalbard, the Antarctic Peninsula, and Continental Antarctica. Results presented separately for Cercozoa, green algae, and ochrophytes. The table displays F-values with degrees of freedom (df) for the nominator and denominator, along with associated p-values derived from one-way ANOVA comparisons.

| ANOVA |  |  |  |  |
| --- | --- | --- | --- | --- |
| Taxon |  | *df* | *F* | *p* |
| Cercozoa | OTU richness | 2, 113 | 10.368 | **7.34e-5** |
|  | Exp(Shannon) | 2, 113 | 8.809 | **0.000278** |
|  | Inverse Simpson | 2, 113 | 4.955 | **0.009** |
| Green algae | OTU richness | 2, 113 | 1.853 | 0.162 |
|  | Exp(Shannon) | 2, 113 | 6.278 | **0.003** |
|  | Inverse Simpson | 2, 113 | 3.888 | **0.023** |
| Ochrophytes | OTU richness | 2, 113 | 4.407 | **0.014** |
|  | Exp(Shannon) | 2, 113 | 3.801 | **0.025** |
|  | Inverse Simpson | 2, 113 | 3.217 | **0.044** |

Values marked in bold indicate statistical significance (p < 0.05).

**Table S12. PERMANOVA results** for taxonomic groups across environmental factors. Analysis of polar biocrusts samples (N=116) from Svalbard, the Antarctic Peninsula, and Continental Antarctica.

| **PERMANOVA** |  |  |  |  |
| --- | --- | --- | --- | --- |
| *Taxon* | *Factor* | *R^2^* | *F* | *p* |
| Cercozoa | Region | 0.1208 | 10.246 | **<0.001** |
|  | pH | 0.1061 | 18.009 | **<0.001** |
|  | Green algae | 0.0348 | 5.903 | **<0.001** |
|  | P (g/kg) | 0.0298 | 5.063 | **<0.001** |
|  | %C | 0.0297 | 5.046 | **<0.001** |
|  | %N | 0.0229 | 3.881 | **<0.001** |
|  | Ochrophytes | 0.0206 | 3.493 | **<0.001** |
|  | CN ratio | 0.0106 | 1.799 | **0.040** |
|  | *Residuals* | 0.6247 |  |  |
| Green algae | Region | 0.1429 | 12.695 | **<0.001** |
|  | pH | 0.0817 | 14.511 | **<0.001** |
|  | Cercozoa | 0.0537 | 9.540 | **<0.001** |
|  | %C | 0.0377 | 6.702 | **<0.001** |
|  | Ochrophytes | 0.0307 | 5.457 | **<0.001** |
|  | P (g/kg) | 0.0281 | 5.002 | **<0.001** |
|  | CN ratio | 0.0165 | 2.929 | **0.006** |
|  | %N | 0.0123 | 2.192 | **0.027** |
|  | *Residuals* | 0.6402 |  |  |
| Ochrophytes | Region | 0.0571 | 3.777 | **<0.001** |
|  | pH | 0.0468 | 6.194 | **<0.001** |
|  | Cercozoa | 0.0224 | 2.961 | **0.004** |
|  | %N | 0.0192 | 2.540 | **0.008** |
|  | Green algae | 0.0169 | 2.240 | **0.016** |
|  | P (g/kg) | 0.0139 | 1.836 | **0.047** |
|  | %C | 0.0123 | 1.636 | 0.077 |
|  | CN ratio | 0.0099 | 1.321 | 0.197 |
|  | *Residuals* | 0.8013 |  |  |

Values marked in bold indicate statistical significance (p < 0.05).

*8. FlashWeave: Network topology indices*

**Table S13. Network topological features,** including inter- and intra-domain co-occurrences for Cercozoa, green algae, and ochrophytes. N=116

|  | Features | *Svalbard* | *An. Peninsula* | *Continental An.* |
| --- | --- | --- | --- | --- |
| Network OTU level | Total node number | 306 | 303 | 135 |
|  | Total edge number | 407 | 422 | 140 |
|  | Inter-domain edges | 89 | 138 | 51 |
|  | Intra-domain edges | 318 | 284 | 89 |
|  | Cercozoa/Endomyxa-algae edges | 72/17.7% | 86/20.4% | 46/32.9% |
|  | Potential predator-prey edges (according to Dumack et al, 2017) | 26/6.4% | 20/4.7% | 13/9.3% |
|  | Average degree | 2.660 | 2.785 | 2.074 |
|  | Average clustering coefficient | 0.013 | 0.001 | 0.018 |
|  | Average shortest path length | 6.698 | 6.047 | 8.075 |
|  | Network density | 0.009 | 0.010 | 0.021 |
| Network aggregated to order level | Total node number | 30 | 28 | 23 |
|  | Total edge number | 157 | 153 | 71 |
|  | Average degree | 10.47 | 10.93 | 6.17 |
|  | Average clustering coefficient | 0.641 | 0.658 | 0.343 |
|  | Average shortest path length | 1.848 | 1.817 | 2.182 |
|  | Network density | 0.257 | 0.286 | 0.194 |

*9. Feeding rate experiments*

**Table S14. Summary of the ANCOVA models for predator counts**.

| **Factor** | ***df*** | **SS** | **MS** | ***F*** | ***p*-value** |
| --- | --- | --- | --- | --- | --- |
| *Cercomonas* grazing on *Auxenochlorella* | | | | | |
| Day | 6 | 58855281605 | 9809213601 | 20.10161335 | 5.79E-09 |
| Control | 1 | 60712284112 | 60712284112 | 124.4151581 | 8.19E-12 |
| Control:Day | 6 | 14451082669 | 2408513778 | 4.935667087 | **0.001473557** |
| Residuals | 28 | 13663479445 | 487981408.7 |  |  |
| *Cercomonas* grazing on *Parietochloris* | | | | | |
| Day | 6 | 9835765566 | 1639294261 | 114.3236789 | 2.27E-18 |
| Control | 1 | 4544600426 | 4544600426 | 316.9384852 | 8.38E-17 |
| Control:Day | 6 | 1411229706 | 235204950.9 | 16.40309243 | **5.16E-08** |
| Residuals | 28 | 401493721.5 | 14339061.48 |  |  |
| *Euglypha* grazing on *Parietochloris* | | | | | |
| Day | 6 | 5255968285 | 875994714.2 | 54.5253969 | 3.69E-14 |
| Control | 1 | 148582160.7 | 148582160.7 | 9.248344942 | 0.005072897 |
| Control:Day | 6 | 237514957.7 | 39585826.28 | 2.463979353 | **0.048586813** |
| Residuals | 28 | 449842704.3 | 16065810.87 |  |  |
| *Euglypha* grazing on *Bracteacoccus* | | | | | |
| Day | 6 | 1479888151 | 246648025.2 | 2.775651985 | 0.030254767 |
| Control | 1 | 4473059148 | 4473059148 | 50.33754271 | 1.02E-07 |
| Control:Day | 6 | 1607550323 | 267925053.9 | 3.015092892 | **0.02114046** |
| Residuals | 28 | 2488116213 | 88861293.32 |  |  |
| *Euglypha* grazing on *Auxenochlorella* | | | | | |
| Day | 6 | 50498264697 | 8416377450 | 15.77906056 | 7.74E-08 |
| Control | 1 | 1.29524E+11 | 1.29524E+11 | 242.8326834 | **2.51E-15** |
| Control:Day | 6 | 1258543297 | 209757216.1 | 0.393253729 | 0.877040559 |
| Residuals | 28 | 14934892210 | 533389007.5 |  |  |
| *Euglypha* grazing on *Stichococcus* | | | | | |
| Day | 6 | 2264766454 | 377461075.6 | 2.754748757 | 0.031223994 |
| Control | 1 | 1274220897 | 1274220897 | 9.299391802 | 0.00496823 |
| Control:Day | 6 | 936673396.3 | 156112232.7 | 1.139322719 | 0.365925722 |
| Residuals | 28 | 3836614896 | 137021960.6 |  |  |
| *Euglypha* grazing on *Pinnularia* | | | | | |
| Day | 6 | 28443957.89 | 4740659.649 | 9.06382308 | 1.54E-05 |
| Control | 1 | 1411467.232 | 1411467.232 | 2.698630617 | 0.111619357 |
| Control:Day | 6 | 1154428.445 | 192404.7408 | 0.367864951 | 0.893068667 |
| Residuals | 28 | 14644865.53 | 523030.9116 |  |  |
| *Fisculla* grazing on *Auxenochlorella* | | | | | |
| Day | 6 | 30474542577 | 5079090429 | 7.222679678 | 9.94E-05 |
| Control | 1 | 2.03121E+11 | 2.03121E+11 | 288.8465246 | 2.76E-16 |
| Control:Day | 6 | 10445655926 | 1740942654 | 2.475693494 | **0.047723428** |
| Residuals | 28 | 19689995731 | 703214133.3 |  |  |
| *Rhogostoma* grazing on *Leptosira* | | | | | |
| Day | 6 | 19697244.03 | 3282874.005 | 1.119509938 | 0.376415631 |
| Control | 1 | 9361925.188 | 9361925.188 | 3.192558797 | 0.08480666 |
| Control:Day | 6 | 12325637.61 | 2054272.934 | 0.700538297 | 0.651469953 |
| Residuals | 28 | 82107776.84 | 2932420.601 |  |  |

**Table S15. Summary of the ANCOVA models for algae counts**.

| **Factor** | ***df*** | **SS** | **MS** | ***F*** | ***p*-value** |
| --- | --- | --- | --- | --- | --- |
| *Cercomonas* grazing on *Auxenochlorella* | | | | | |
| Day | 3 | 154277550.5 | 51425850.15 | 54.60122053 | 1.26E-08 |
| Control | 1 | 313080937 | 313080937 | 332.4126142 | 3.97E-12 |
| Control:Day | 3 | 153040539.8 | 51013513.26 | 54.16342325 | **1.34E-08** |
| Residuals | 16 | 15069509.33 | 941844.3333 |  |  |
| *Cercomonas* grazing on *Parietochloris* | | | | | |
| Day | 3 | 24870393 | 8290131 | 9.088187 | 0.001363 |
| Control | 1 | 42559538 | 42559538 | 46.65657 | 8.19E-06 |
| Control:Day | 2 | 18622961 | 9311481 | 10.20786 | **0.001843** |
| Residuals | 14 | 12770625 | 912187.5 |  |  |
| *Euglypha* grazing on *Parietochloris* | | | | | |
| Day | 5 | 13995.58333 | 2799.116667 | 34.39187713 | 3.45E-10 |
| Control | 1 | 18000.69444 | 18000.69444 | 221.168942 | 1.31E-13 |
| Control:Day | 5 | 16437.13889 | 3287.427778 | 40.3916041 | **6.40E-11** |
| Residuals | 24 | 1953.333333 | 81.38888889 |  |  |
| *Euglypha* grazing on *Bracteacoccus* | | | | | |
| Day | 5 | 235.5833333 | 47.11666667 | 4.167567568 | 0.007232903 |
| Control | 1 | 448.0277778 | 448.0277778 | 39.62899263 | 1.66E-06 |
| Control:Day | 5 | 731.8055556 | 146.3611111 | 12.94594595 | **3.62E-06** |
| Residuals | 24 | 271.3333333 | 11.30555556 |  |  |
| *Euglypha* grazing on *Auxenochlorella* | | | | | |
| Day | 5 | 244.9166667 | 48.98333333 | 4.343349754 | 0.005891103 |
| Control | 1 | 684.6944444 | 684.6944444 | 60.71182266 | 5.02E-08 |
| Control:Day | 5 | 706.4722222 | 141.2944444 | 12.52857143 | **4.75E-06** |
| Residuals | 24 | 270.6666667 | 11.27777778 |  |  |
| *Euglypha* grazing on *Stichococcus* | | | | | |
| Day | 5 | 69.13888889 | 13.82777778 | 1.575316456 | 0.204956781 |
| Control | 1 | 250.6944444 | 250.6944444 | 28.56012658 | 1.74E-05 |
| Control:Day | 5 | 93.13888889 | 18.62777778 | 2.122151899 | 0.097444559 |
| Residuals | 24 | 210.6666667 | 8.777777778 |  |  |
| *Euglypha* grazing on *Pinnularia* | | | | | |
| Day | 5 | 207.1388889 | 41.42777778 | 4.131301939 | 0.007548441 |
| Control | 1 | 318.0277778 | 318.0277778 | 31.71468144 | 8.49E-06 |
| Control:Day | 5 | 659.1388889 | 131.8277778 | 13.14626039 | **3.18E-06** |
| Residuals | 24 | 240.6666667 | 10.02777778 |  |  |
| *Fisculla* grazing on *Auxenochlorella* | | | | | |
| Day | 3 | 23802.45833 | 7934.152778 | 4.960654058 | 0.012716939 |
| Control | 1 | 107334.375 | 107334.375 | 67.108451 | 4.07E-07 |
| Control:Day | 3 | 25331.45833 | 8443.819444 | 5.279311902 | **0.010092686** |
| Residuals | 16 | 25590.66667 | 1599.416667 |  |  |
| *Rhogostoma* grazing on *Leptosira* | | | | | |
| Day | 3 | 7617714 | 2539238 | 8.55482 | 0.001281 |
| Control | 1 | 9538204 | 9538204 | 32.13469 | 3.49E-05 |
| Control:Day | 3 | 7709935 | 2569978 | 8.658386 | **0.001209** |
| Residuals | 16 | 4749113 | 296819.5 |  |  |

*
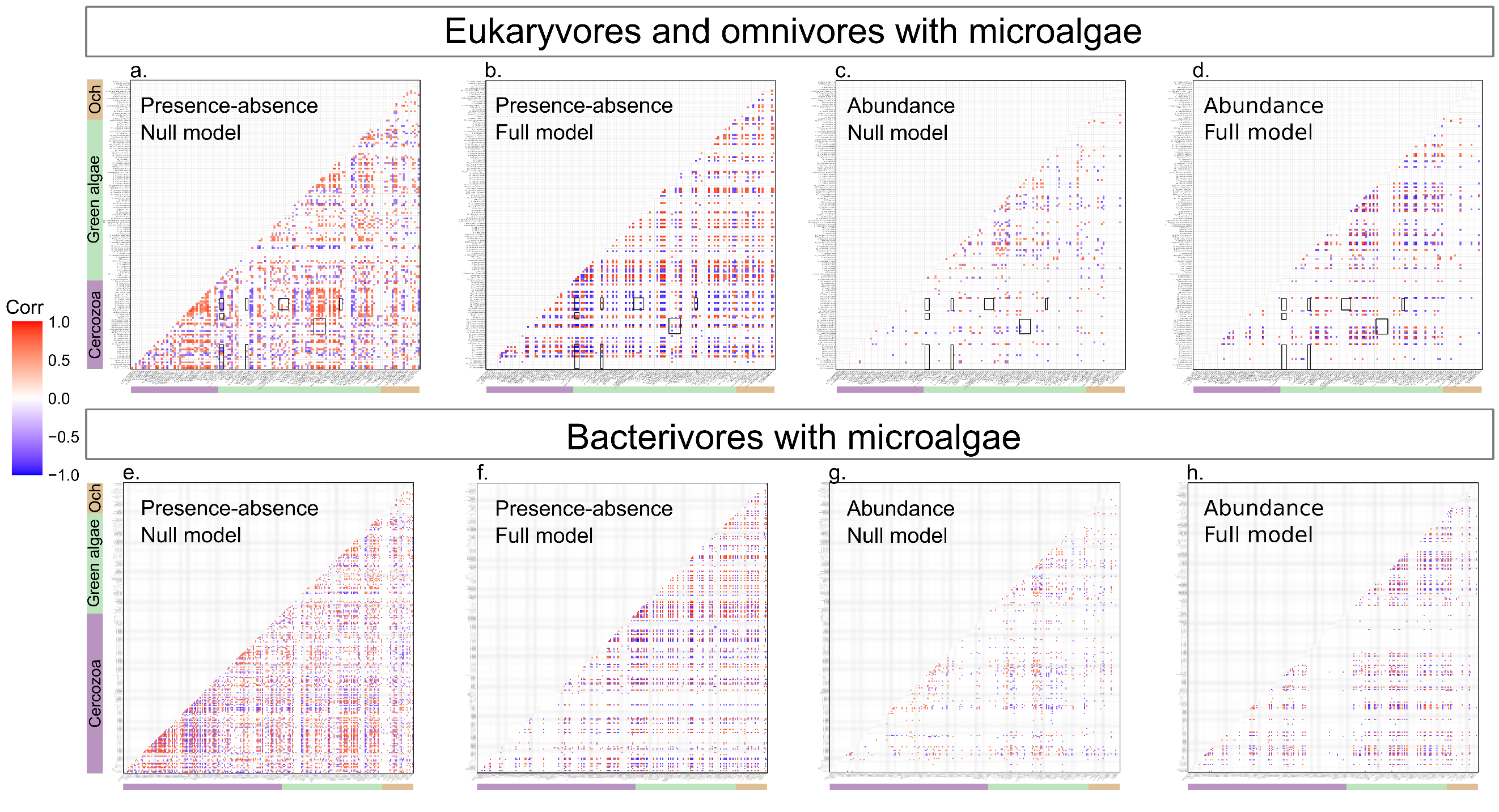
10. HMSC networks and calculations*

**Figure S9.** **HMSC species-to-species** **co-occurrence and correlation networks for Cercozoan algivores and bacterivores with microalgae, inferred using presence-absence and abundance models.** Panels (a–d) depict associations between cercozoan eukaryvores and omnivores with green algae and ochrophytes, while panels (e–h) show associations between cercozoan bacterivores and microalgae. The presence-absence models (a, b, e, f) were inferred using a probit model, whereas the abundance models (c, d, g, h) were based on a normal model. Each model includes both a null version (a, c, e, g) to represent random patterns and a full version (b, d, f, h) that incorporates environmental covariates. The correlations marked with black squares represent interactions validated through laboratory testing.

**Table S16. Summary of co-occurrence and correlation interactions identified in Cercozoan-algae HMSC networks across models**

| Model | Cercozoa trait | Type of association | Positive | Negative | Total (subnetwork) | Total (model) | % Potential predatory |
| --- | --- | --- | --- | --- | --- | --- | --- |
| modpa_null | Eukaryvores | Total | 3895 | 2338 | 6233 | 21689 | 6.777629213 |
|  | Eukaryvores | Potential predatory | 836 | 634 | 1470 |  |  |
|  | Bacterivores | Total | 9158 | 6298 | 15456 |  |  |
|  | Bacterivores | Cercozoa-algae | 2013 | 1486 | 3499 |  |  |
| modpa_full | Eukaryvores | Total | 2125 | 1686 | 3811 | 10515 | **7.522586781** |
|  | Eukaryvores | Potential predatory | 326 | 465 | 791 |  |  |
|  | Bacterivores | Total | 3654 | 3050 | 6704 |  |  |
|  | Bacterivores | Cercozoa-algae | 746 | 872 | 1618 |  |  |
| modabu_null | Eukaryvores | Total | 699 | 512 | 1211 | 4215 | 3.534994069 |
|  | Eukaryvores | Potential predatory | 73 | 76 | 149 |  |  |
|  | Bacterivores | Total | 1694 | 1310 | 3004 |  |  |
|  | Bacterivores | Cercozoa-algae | 339 | 356 | 695 |  |  |
| modabu_full | Eukaryvores | Total | 905 | 788 | 1693 | 5573 | **4.790956397** |
|  | Eukaryvores | Potential predatory | 135 | 132 | 267 |  |  |
|  | Bacterivores | Total | 2068 | 1812 | 3880 |  |  |
|  | Bacterivores | Cercozoa-algae | 460 | 446 | 906 |  |  |

Overview of interactions inferred between Cercozoa and microalgae using Hierarchical Modeling of Species Communities (HMSC) with null and full models for both presence-absence (modpa) and abundance (modabu) data. Interactions are categorized by Cercozoa traits (eukaryvores or bacterivores) and type of association (total, Cercozoa-algae subnetwork, and potential predatory interactions).

Supplementary references

1. Jensen Ø. The Svalbard Treaty and Norwegian Sovereignty. Arct Rev Law Polit. 2020;11:82–107.

2. Adakudlu M, Andersen J, Bakke J, Beldring S, Benestad R, Bilt W van der, et al. Climate in Svalbard 2100 – a knowledge base for climate adaptation. Norwegian Centre for Climate Services (NCCS) for Norwegian Environment Agency (Miljødirektoratet); 2019. https://repository.oceanbestpractices.org/handle/11329/1382

3. Norwegian Meteorological Institute. Annual precipitation in Svalbard, Hopen, Bjørnøya and Jan Mayen, filtered. MOSJ. 2022. https://mosj.no/en/indikator/climate/atmosphere/air-temperature-and-precipitation/

4. Burnett HA, Bieker VC, Le Moullec M, Peeters B, Rosvold J, Pedersen ÅØ, et al. Contrasting genomic consequences of anthropogenic reintroduction and natural recolonization in high‐arctic wild reindeer. Evol Appl. 2023;eva.13585.

5. Johansen BE, Karlsen SR, Tømmervik H. Vegetation mapping of Svalbard utilising Landsat TM/ETM+ data. Polar Rec. 2012;48:47–63.

6. Brožová V, Bolstad JS, Seregin AP, Eidesen PB. From everywhere all at once: Several colonization routes available to Svalbard in the early Holocene. Ecol Evol. 2023;13:e9892.

7. Øvstedal D, Tønsberg T, Elvebakk A. The lichen flora of Svalbard. Sommerfeltia. 2009;33:3–393.

8. Prestø T, Lueth M, Hassel K. Bryophytes of the Longyearbyen area. 2014.

9. Borchhardt N, Baum C, Mikhailyuk T, Karsten U. Biological Soil Crusts of Arctic Svalbard—Water Availability as Potential Controlling Factor for Microalgal Biodiversity. Front Microbiol. 2017;8:1485.

10. Rippin M, Lange S, Sausen N, Becker B. Biodiversity of biological soil crusts from the Polar Regions revealed by metabarcoding. FEMS Microbiol Ecol. 2018. https://doi.org/10.1093/femsec/fiy036

11. Borchhardt N, Schiefelbein U, Abarca N, Boy J, Mikhailyuk T, Sipman HJM, et al. Diversity of algae and lichens in biological soil crusts of Ardley and King George islands, Antarctica. Antarct Sci. 2017;29:229–37.

12. Garrido-Benavent I, Pérez-Ortega S, Durán J, Ascaso C, Pointing SB, Rodríguez-Cielos R, et al. Differential Colonization and Succession of Microbial Communities in Rock and Soil Substrates on a Maritime Antarctic Glacier Forefield. Front Microbiol. 2020;11.

13. Boy J, Godoy R, Shibistova O, Boy D, McCulloch R, de la Fuente AA, et al. Successional patterns along soil development gradients formed by glacier retreat in the Maritime Antarctic, King George Island. Rev Chil Hist Nat. 2016;89:6.

14. Michel R, Schaefer C, López-Martínez J, Simas F, Haus N, Serrano E, et al. Soils and landforms from Fildes Peninsula and Ardley Island, Maritime Antarctica. Geomorphology. 2014;225:76–86.

15. Pasik M, Bakuła K, Różycki S, Ostrowski W, Kowalska M, Fijałkowska A, et al. Glacier Geometry Changes in the Western Shore of Admiralty Bay, King George Island over the Last Decades. Sensors. 2021;21:1532.

16. Henriques DK, Silva BGC, Zuñiga GE, Câmara PEAS. Contributions to the bryological knowledge of ASPA 125, Fildes Peninsula, King George Island. Biol Res. 2018;51:29.

17. Andreyev MP. The lichens in the vicinity of Bellingshausen station, King George Island. Polar Geogr Geol. 1989;13:42–5.

18. So JE, Halda JP, Hong SG, Hur J-S, Kim JH. The Revision of Lichen Flora Around Maxwell Bay, King George Island, Maritime Antarctic. J Microbiol. 2023;61:159–73.

19. Lukashanets DA, Convey P, Borodin OI, Miamin VY, Hihiniak YH, Gaydashov AA, et al. Eukarya biodiversity in the Thala Hills, East Antarctica. Antarct Sci. 2021;33:605–23.

20. Ohtani S, Suyama K, Yamamoto H, Aridomi Y. Distribution of soil algae at the monitoring sites in the vicinity of Syowa Station between austral summers of 1992/1993 and 1997/1998. 2000;

21. Hall J, Fu K, Lo C, Lewis LA, Karol KG. An assessment of proposed DNA barcodes in freshwater green algae. Cryptogamie. 2010;31:529–55.

22. Guo L, Sui Z, Zhang S, Ren Y, Liu Y. Comparison of potential diatom ‘barcode’ genes (the 18S rRNA gene and ITS, COI, rbcL) and their effectiveness in discriminating and determining species taxonomy in the Bacillariophyta. Int J Syst Evol Microbiol. 2015;65:1369–80.

23. Visco JA, Apothéloz-Perret-Gentil L, Cordonier A, Esling P, Pillet L, Pawlowski J. Environmental Monitoring: Inferring the Diatom Index from Next-Generation Sequencing Data. Environ Sci Technol. 2015;49:7597–605.

24. Van Borm S, Boomsma JJ. Group-specific polymerase chain reaction amplification of SSU rRNA-encoding gene fragments from 12 microbial taxa. Mol Ecol Notes. 2002;2:356–9.

25. Bradley IM, Pinto AJ, Guest JS. Design and Evaluation of Illumina MiSeq Compatible Primers for the 18S rRNA Gene for Improved Characterization of Mixed Phototrophic Communities. 2016;16.

26. Lee S-R, Oak JH, Chung IK, Lee JA. Effective molecular examination of eukaryotic plankton species diversity in environmental seawater using environmental PCR, PCR-RFLP, and sequencing. J Appl Phycol. 2010;9.

27. Stoeck T, Bass D, Nebel M, Christen R, Jones MDM, Breiner H-W, et al. Multiple marker parallel tag environmental DNA sequencing reveals a highly complex eukaryotic community in marine anoxic water. Mol Ecol. 2010;19:21–31.

28. Zimmermann J, Jahn R, Gemeinholzer B. Barcoding diatoms: evaluation of the V4 subregion on the 18S rRNA gene, including new primers and protocols. Org Divers Evol. 2011;11:173–92.

29. Klindworth A, Pruesse E, Schweer T, Peplies J, Quast C, Horn M, et al. Evaluation of general 16S ribosomal RNA gene PCR primers for classical and next-generation sequencing-based diversity studies. Nucleic Acids Res. 2013;41:e1–e1.

30. Fiore-Donno AM, Rixen C, Rippin M, Glaser K, Samolov E, Karsten U, et al. New barcoded primers for efficient retrieval of cercozoan sequences in high-throughput environmental diversity surveys, with emphasis on worldwide biological soil crusts. Mol Ecol Resour. 2018;18:229–39.
